# Dietary Diversity, Iron Status, and Anaemia Among Adivasi Women: Insights from the chiguru Cohort in Chamarajanagar District, Southern Karnataka

**DOI:** 10.12688/wellcomeopenres.24846.1

**Published:** 2025-10-29

**Authors:** Maithili Karthik, Jai Prabhakar Sosale Chandrashekaraswamy, Prafulla Shriyan, Suresh Shapeti, Prashanth Thankachan, Tanya Seshadri, Giridhara R Babu

**Affiliations:** 1 Public Health, Indian Institute of Public Health, Bengaluru, Karnataka, 560038, India; 2 Anthropology, Karnatak University Dharwad, Dharwad, Karnataka, 580003, India; 3 Anthropology, Centre for Multi-disciplinary Development Research (CMDR), Dharwad, Karnataka, 580004, India; 4 Division of Nutrition, St. John’s Research Institute, Bengaluru, Karnataka, 560034, India; 5 Centre of Adivasi Health, Institute of Public Health Bengaluru, Bengaluru, Karnataka, 560070, India; 6 Population Medicine, College of Medicine, QU Health, Qatar University College of Medicine, Doha, Doha, Qatar

**Keywords:** anaemia; dietary diversity; soluble transferrin receptor; adivasi women; iron deficiency; tribal health

## Abstract

**Introduction:**

Anaemia remains a significant public health concern, particularly among marginalized populations such as tribal communities. This study examines the complex relationships between dietary diversity, iron status biomarkers, and anaemia prevalence among Adivasi women in southern Karnataka, India.

**Method:**

A cross-sectional analysis using data from the chiguru Adivasi cohort in Chamarajanagar District, Karnataka, India. The study included 479 women, primarily from the Soliga tribe. The authors assessed HHDD scores, measured haemoglobin, sTfR levels, and ferritin, and collected sociodemographic data. The authors then performed multivariable linear and logistic regression analyses to examine associations between these variables.

**Results:**

The HHDD scores did not directly predict haemoglobin levels but were negatively associated with sTfR levels ( *β* = -2.975, *p* = 0.045). After adjusting for confounders , sTfR emerged as a crucial predictor of anaemia, with elevated levels considerably increasing the odds of anaemia (OR = 1.68, *p* < 0.01). Geographic variations in dietary diversity among the Soliga community are influenced by forest access, seasonal availability of wild foods, and local agricultural practices.

**Conclusions:**

This study highlights the need for cost-effective, culturally relevant interventions to address anaemia among Adivasi women. Promoting daily consumption of locally available iron-rich foods, including green leafy vegetables, and encouraging community-led vegetable cultivation can enhance dietary diversity. Strengthening nutritional awareness programs and improving participation through community engagement will further support sustainable dietary practices. Integrating these efforts into broader public health initiatives can effectively reduce anaemia and improve overall well-being in tribal populations.

## Introduction

Anaemia remains a critical global health issue and has a disproportionate impact on tribal communities. Globally, an estimated 1.62 billion people are affected by anaemia, with prevalence rates as high as 75% in specific tribal populations ^1^. Anaemia in pregnancy is associated with severe health implications, including impaired cognitive and physical performance, increased morbidity, and adverse maternal and child health outcomes ^2^. For example, it increases the risk of low birth weight by 29% and preterm birth by 21% ^3^. Despite the multifactorial aetiology of anaemia in tribal communities, iron deficiency is the predominant cause due to nutritional, socioeconomic, and cultural factors. This is exacerbated by poor dietary intake and diets rich in iron absorption inhibitors. A study found that 68.5% of tribal women in West Bengal, India, had inadequate iron intake, with 89.2% consuming diets high in phytates and polyphenols. Socioeconomic marginalisation, geographical isolation, and limited access to healthcare resources further compound the issue ^4^.

Dietary diversity is a key determinant of nutritional adequacy and is particularly relevant in tribal populations, where diets often consist of staple foods with limited variety ^5^. Consuming a diverse diet rich in iron-containing foods and other essential micronutrients is associated with better health outcomes, including reduced risk of anaemia ^6^. However, evidence linking dietary diversity to anaemia among tribal populations is sparse. Studies have shown that tribal communities often have diets dominated by cereals and starchy staples. Low consumption of dairy products, fruits, and vegetables leads to inadequate intake of key nutrients like iron, calcium, and vitamin A ^7^. Inadequate intakes of dairy foods, fruits, and vegetables lead to inadequate intakes of key nutrients, such as iron, calcium, and vitamin A. Protein also plays a role in iron metabolism. Animal proteins like meat, poultry, and fish contain high amounts of heme iron and facilitate the absorption of non-heme iron through the stimulation of gastric acid secretion and the provision of amino acids that deliver soluble iron to the body. Legume and soy proteins tend to suppress the absorption of iron due to their phytate composition ^8^. This limited dietary diversity contributes to high rates of anaemia and other micronutrient deficiencies in these populations.

Despite being an acute-phase reactant, ferritin is currently the best available biomarker for assessing iron stores, as recommended by the WHO. While its levels can be influenced by inflammation and infection, adjustments based on CRP and AGP are ideal but were not feasible in the present study due to logistical limitations. The potential influence of hemodilution in pregnancy and obesity is acknowledged as a confounder, though the interpretation of iron status has been made cautiously within this context ^9^. In contrast, sTfR is a reliable marker of tissue iron deficiency, which rises earlier in pregnancy and remains unaffected by inflammation or infection. The sTfR is a sensitive indicator of iron status and iron-deficiency anaemia ^10^, reflects tissue-level iron demand and may be an early marker of iron deficiency, even in individuals with normal systemic iron parameters. The sTfR index, calculated as the ratio of sTfR to the logarithm of ferritin, has been shown to improve the detection of iron deficiency anaemia compared to other markers ^11, 12^.

While iron biomarkers are helpful, they are only part of the picture of anaemia, particularly in marginalized or tribal populations, where there is a plethora of nutritional, infectious, and environmental factors. Field research has demonstrated that food diversity, as a measure of micronutrients alongside iron intake, is a better predictor of anaemia than single-nutrient biomarkers in isolation ^13, 14^. Most existing studies focus either on dietary patterns or biochemical markers in isolation, failing to capture their interplay. This gap is particularly pronounced in tribal populations, where unique cultural, dietary, and environmental factors may influence anaemia prevalence differently than in general populations. For instance, tribal communities often face limited access to nutritious foods, including iron, folate, and vitamin B12, which can contribute to higher anaemia rates ^15, 16^. Additionally, cultural norms, poor sanitation practices, and insufficient maternal care may collectively impact anaemia prevalence among women of reproductive age in these communities ^17^. Addressing this research gap is crucial for designing targeted interventions that are culturally and contextually appropriate, as the prevalence of anaemia in some tribal populations has been reported to be as high as 89–96.5% ^18^. By combining dietary diversity assessment with iron biomarker analysis, the authors aim to understand the aetiology of anaemia in tribal populations ^19^. By exploring the relationship between nutritional diversity and iron biomarkers, the research aims to identify significant predictors of anaemia and deepen the understanding of anaemia in tribal populations. This can inform the design of targeted interventions that consider these populations’ distinctive needs and contexts, potentially leading to more effective anaemia prevention and control in vulnerable populations. We hypothesize that increased household dietary diversity (HHDD) is associated with improved iron status, measured as decreased soluble transferrin receptor (sTfR) levels and decreased risk of iron deficiency anaemia (IDA) in Adivasi women. The association remains strong even after adjusting for the key sociodemographic and nutritional confounding factors.

## Objective

We examined the association between dietary diversity and iron deficiency anaemia (IDA) using soluble transferrin receptor (sTfR) levels and investigated potential risk factors for IDA in the tribal cohort.

## Methodology

### Study setting

This is a cross-sectional study of Adivasi women conducted in the chiguru Adivasi family cohort living in the Chamrajanagar district of Southern Karnataka ^20^. As per NFHS-5, the prevalence of anaemia is 46.3% in Chamrajanagar District. To determine the statistical power of the sample size (479) for estimating the prevalence of anaemia (46.3%) in Chamrajanagar District with a marginal error of 4.47% and a design effect of 1, the authors use standard power calculation methods for proportion estimation at a 95% confidence level (Z-score = 1.96, which seems to be a typographical error). Based on the formula for power calculation, the sample size of 479 achieves approximately 80% power, ensuring a reliable estimation of anaemia prevalence within the given confidence interval.

**Figure 1.**
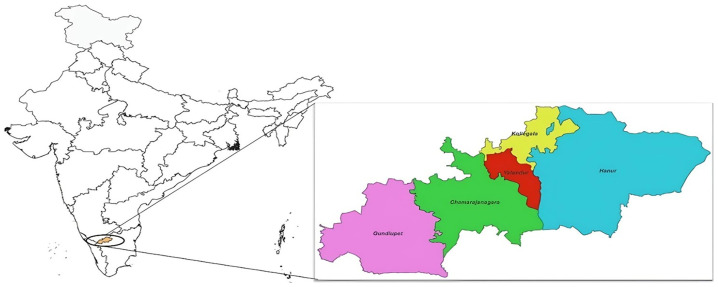
Chamrajanagar district study area.

**Figure 2.**
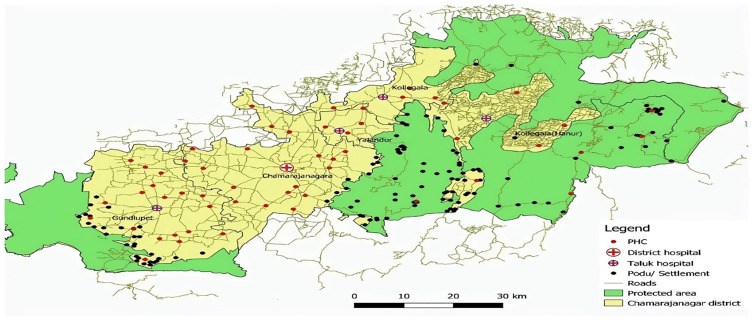
The black dots represent the settlements of both Adivasi communities within and around the protected areas (Green).

The study was conducted in the Chamarajanagar district, Karnataka ( Figure 1), situated in the southern part of Karnataka between Tamil Nadu and Kerala, is nestled in the leeward region of the Nilgiris mountain range. Approximately 48% of its total land area is covered by forests, with substantial portions designated as regions protected under the Wildlife Protection Act of 1972. Notable among these are the Bandipur and Biligiriranga Hills (B.R. Hills) tiger reserves, in addition to the Malai Mahadeshwara (M.M. Hills) wildlife sanctuary. In the hilly terrain and surrounding areas of Chamrajanagar, three prominent tribal communities—Soliga, Jenu Kuruba, and Kadu Kuruba—reside in distinct Podus. These communities have established unique habitats within this region, contributing to the diverse cultural landscape. The Podus are unique settlement clusters that serve as the primary living spaces for different Soliga tribal groups. The Soligas are a scheduled tribe largely found in the Biligiri Rangaswamy Temple (BRT) Tiger Reserve and its fringes in southern Karnataka. Their major activities are subsistence farming, minor forest products (honey and amla, especially), gathering, and wage labor. The Soliga language (Dravidian) is the spoken language, and the people typically live in dispersed villages called Podus, with 20–200 persons per Podu ^21^. The majority of the families are of low socioeconomic status and have limited health and education services ^22^. ( Figure 2) The black dots represent the settlements of both Adivasi communities within and around the protected areas (Green). Each Podu functions as a self-sufficient unit, fostering a strong sense of community, cultural identity, and traditional practices. These settlements are typically located in forested regions, reflecting the Soligas’ deep connection with nature and their sustainable way of life. The Podu system plays a crucial role in preserving their heritage, social structure, and Indigenous knowledge across generations of Chamrajanagar. The geographical setting and cultural diversity of these tribal communities contribute to the area’s unique identity and heritage.

### Inclusion and exclusion criteria

The research targeted participants who live in and around Chamrajanagar and the surrounding talukas, focusing on tribal populations like the Soliga, Jenu Kuruba, and Kadu Kuruba. The inclusion criteria are postnatal women and their female relatives over 18 years of age who are willing to consent to baseline data collection, anthropometric measurements, and blood sampling. They are also enrolled in the chiguru Adivasi family cohort. Exclusion criteria encompass non-tribal individuals who do not consent to participate will be excluded from the research.

### Informed consent

Only women aged 18 years and above were enrolled in this study; no minors participated. Written informed consent was obtained from all participants before data collection. For illiterate participants, the consent process was explained orally in the local language, and their consent was documented using a thumbprint in the presence of a literate witness. This approach was used instead of written consent to ensure inclusivity and ethical participation of individuals unable to read or write.

### Data collection

The HHDD Score is the primary exposure variable in this study, assessing dietary diversity among tribal women. It is derived from a validated FFQ that categorizes food groups based on international standards, such as the FAO and HDDS classifications. Participants report their consumption of these food groups within a specified recall period (e.g., 24 h to 7 days) ^23^. The HDDS is calculated by summing the number of food groups consumed, with higher scores indicating better dietary diversity and nutrition, while lower scores reflect food insecurity and poor diet quality.

Dietary diversity is particularly significant in tribal populations due to the influence of traditional food habits, seasonal availability, and socioeconomic factors. HDDS provides a quantitative measure of dietary practices and their potential link to anaemia. It was collected from postnatal mothers through a self-reported questionnaire at the study’s initiation phase to capture early postpartum dietary patterns. HDDS has demonstrated strong internal consistency and repeatability ^24^. The Household Dietary Diversity Score (HDDS) has shown internal consistency and has been successfully applied across many different cultures, even in the case of tribal societies with diversified and localized food systems. The HDDS has demonstrated strong internal consistency and high inter-rater reliability in studies conducted in India ^23^. Additionally, the HDDS has been validated by the FAO as a predictor of food security, with an area under the ROC curve AUC of 0.89, indicating high predictive accuracy ^25^. Additionally, its reliability across recall periods has been confirmed, with no significant differences found between 24-h and 7-day recall periods ( *p* > 0.05) ^26^.

In the study, iron storage was assessed using sTfR; however, as sTfR reflects iron deficiency rather than storage, the authors now incorporate ferritin for a more appropriate evaluation. Which has a dual role in reflecting iron metabolism and, as a predictor variable, specifically targets iron deficiency anaemia rather than anaemia of other etiologies. Phlebotomists prepared and collected blood samples using standardized processes. With informed consent, biological samples were obtained from individuals at the PODUs by trained phlebotomists at B.R. Hills. The samples were processed at the VGKK laboratory, subsequently transported to the Vailes biorepository, sealed with Parafilm, and cryo-labels preserved at −80 °C for later analysis of sTfR and ferritin levels.to maintain their integrity. Temperature checks were conducted, and meticulous sample recording was done in the logbook.

### Laboratory analysis

Haemoglobin (Hb), mean corpuscular volume (MCV), and red blood cell (RBC) count were measured using the Sysmex XN-1000 automated haematology analyser (Sysmex Corporation, Kobe, Japan). Serum ferritin was analysed using the Chemiluminescent Microparticle Immunoassay (CMIA) kit (Abbott Diagnostics, Cat# 7K60-20, Abbott Laboratories, USA). Soluble transferrin receptor (sTfR) was measured using the Human sTfR ELISA kit (R&D Systems, Minneapolis, USA; Cat# DTR100). Quality control was performed using Randox QC materials (Randox Laboratories, Crumlin, UK; Cat# HE1532). All assays and reagents were used according to the manufacturer’s instructions.

Using a DAG, the authors included covariates to adjust, which included sociodemographic factors (age, marital status, tribe or subtribe membership, household size, and education level), economic and household factors, household social living index (SLI), occupation of the participants, source of drinking water, and availability of sanitation facilities), parity (pregnancy history), menstrual history, prevalence of infection (malaria and parasitic infection), BMI, and other anthropometric measures. Demographic, socioeconomic, and clinical data were collected through structured questionnaires and examinations. Anthropometric measurements, including height and weight, were recorded. Blood samples were collected for haemoglobin, red blood cell count, mean corpuscular volume, soluble transferrin receptor, and ferritin analyses.

Trained in both anthropometric assessment and phlebotomy, the team conducted body measurements, collected blood samples, and performed anthropometric measurements on postnatal mothers and other women of the family using calibrated instruments. The team uses a Seca adult stadiometer and weighing machine for height and weight measurements. The weighing machine is calibrated every month with the use of 10 kg butt weight. The stadiometer is calibrated with calibration rods before starting to measure. Each weighing session will have instruments calibrated to ensure accurate measurements. The height is measured to the nearest 0.1 cm by the Seca Hamburg, Germanystadiometer, and the participant stands upright, barefoot, and is positioned according to the Frankfurt plane. Weight is measured to the nearest 0.1 kg (Seca GmBH & Co Kg, Hamburg, Germany) with participants in light clothing and no shoes. Measurements are typically taken two times, and the average is used for accuracy.

### Statistical analysis

Statistical analyses were performed using Stata, version 18. Descriptive statistics summarised demographic, socioeconomic, and clinical characteristics. Chi-square tests were used for categorical variables, while *t*-tests or non-parametric equivalents were used for continuous variables. The normality of the continuous variable was assessed using the Shapiro–Wilk test. Since several variables, including sTfR and ferritin, were not normally distributed ( *p* < 0.05), non-parametric tests (e.g., Wilcoxon rank-sum test) were used where appropriate. Multivariable linear regression assessed the association between haemoglobin levels and predictors, including the HHDD score. A second multivariable regression analysis was performed to assess the association between the sTfR/ferritin ratio—a composite biomarker reflecting iron status—and the dependent variable while controlling for additional covariates. Logistic regression evaluated the relationship between anaemia (outcome) and sTfR. Model fit was assessed using metrics such as the Akaike Information Criterion (AIC) and Bayesian Information Criterion (BIC), with significance set at *p* < 0.05.

## Results

The study included 479 Adivasi women, primarily from the Soliga tribe, with a mean age of 30.91 years (SD = 11.229). The descriptive statistics are provided in Table 1. Most women were young, with a mean age of 30.9 years (SD = 11.229), with a mean haemoglobin (Hb) level of 11.425 g/dL (SD = 1.709). Red blood cell count (RBC) averaged 4.9 million cells/μL (SD = 0.609), while the mean corpuscular volume (MCV) averaged 70.543 fL (SD = 9.093). Soluble transferrin receptor (sTfR) levels were highly variable, with a mean of 10.432 mg/L (SD = 65.134) and a range of 0.72 to 966 mg/L. Similarly, iron ferritin levels (Ferritin) were also highly variable, with a mean of 56.259 μg/dL (SD = 61.689). Other anthropometric and household characteristics include a mean household dietary diversity (HHDD) score of 8.575 (SD = 2.978) among 327 households, average weight (mean weight) of 43.778 kg (SD = 9.588), and average height (mean height) of 149.492 cm (SD = 15.04). The study used this information as one of the variables in the analysis of factors associated with anaemia and dietary diversity among Adivasi women. Among the categorical variables, 69.5% of participants reported education levels less than PUC (Education), and 73.3% were homemakers. Most women had 1–2 pregnancies (35.2% with one and 38.8% with two), while parity (Parity) followed a similar trend, with 40.7% reporting one live birth and 37.9% reporting two. Abortions were uncommon (9.78%), with 90.2% reporting none. Stillbirths were also rare (7.95%), with 92.0% reporting none. Regarding live births, most participants reported one or two (43.1% and 39.1%, respectively).

**Table 1.** Characteristics of the Sample Included in the Study.

Variable	N	Mean ± SD or n (%)
Age (Years)	479	30.91 ± 11.2
Haemoglobin (Hb) g/dL	313	11.42 ± 1.7
Red Blood Cells (RBC) 10⁶/µL	313	4.95 ± 0.6
Mean Corpuscular Volume (MCV) fL	311	70.54 ± 9.0
Serum Transferrin Receptor mg/l (sTfR)	218	10.43 ± 65.13
Ferritin ng/ml	218	56.25 ± 61.68
Weight kg	473	43.77 ± 9.58
Height cm	473	149.49 ± 15.04
Household Dietary Diversity (HHDD) score	327	8.57 ± 2.97
Education - No formal education	333	69.5%
Education - Formal education	146	30.5%
Occupation - Others	128	26.7%
Occupation - Housewife	351	73.3%
Gravidity - 1	115	35.2%
Gravidity - 2	127	38.8%
Gravidity - 3	62	19.0%
Gravidity - 4	18	5.5%
Gravidity - 5	4	1.2%
Gravidity - 6	1	0.3%
Parity - 1	133	40.7%
Parity - 2	124	37.9%
Parity - 3	54	16.5%
Parity - 4	11	3.4%
Parity - 5	4	1.2%
Parity - 6	1	0.3%
Abortion - 0	295	90.2%

The mean HHDD score was 8.575 (SD = 2.978), indicating moderate food diversity among the households surveyed. All households (100%) reported consuming cereals, reflecting their role as a dietary staple. The majority also consumed white roots and tubers (88.4%), vitamin A-rich vegetables and tubers (76.1%), and vitamin A-rich fruits (63.9%). Organ meats were consumed by 66.1% of households, while 74.0% reported egg consumption. Fish consumption was reported by 52.9% of households, and legumes, nuts, and seeds were consumed by 72.5%. Milk and milk products were reported by 51.7% of households, reflecting lower levels of dairy consumption compared to other food groups. Oil and fats (70.0%), sweets (70.9%), and spices, condiments, and beverages (70.9%) were also widely consumed, indicating a broad inclusion of diverse food items in the diet. ( Table 2).

**Table 2.** Composition of the HHDD Score.

Component	Score/ Category	Frequency (Percentage)
HHDD Score	8.575 (2.978)	-
Cereals	Yes	327 (100.0%)
White tubers and roots	No	38 (11.6%)
	Yes	289 (88.4%)
Vegetables ¹	No	78 (23.9%)
	Yes	249 (76.1%)
Fruits ²	No	118 (36.1%)
	Yes	209 (63.9%)
Meat ³	No	111 (33.9%)
	Yes	216 (66.1%)
Eggs	No	85 (26.0%)
	Yes	242 (74.0%)
Fish and other seafood	No	154 (47.1%)
	Yes	173 (52.9%)
Legumes, nuts and seeds	No	90 (27.5%)
	Yes	237 (72.5%)
Milk and milk products	No	158 (48.3%)
	Yes	169 (51.7%)
Oils and fats	No	98 (30.0%)
	Yes	229 (70.0%)
Sweets	No	95 (29.1%)
	Yes	232 (70.9%)
Spices, condiments, and beverages	No	95 (29.1%)
	Yes	232 (70.9%)

¹ The vegetable food group combines vitamin A-rich vegetables and tubers, dark green leafy vegetables, and other vegetables. ² The fruit group is a combination of vitamin A-rich fruits and other fruits. ³ The meat group is a combination of organ meat and flesh meat.

Most participants in both groups were from the Soliga tribe, with no significant differences in tribal distribution ( *p* = 0.35). Marital status, educational status, and BMI were also similar across groups, with no significant differences observed. Additionally, health-related metrics such as Hb, MCV, and ferritin showed no significant differences ( *p* > 0.1). Significant differences were noted in the distribution of participants across taluks ( *p* < 0.001), with a higher proportion of individuals residing in Chamarajanagar (32.3%) and Yelandur (32.3%) having higher food diversity, while those who resided in Hanur had lower food diversity (63.4%). Blood relations also differed significantly ( *p* < 0.001), with more participants in the lower diversity group reporting “Another blood relative” (30.8%) or “Second cousin” (36.9%) compared to the “diversity = 0” group. Furthermore, the median sTfR was significantly higher in the group with lower food diversity (5.23) than in the more diverse group (4.49, *p* = 0.041) ( Table 3).

**Table 3.** Characteristics of the population based on low diversity (less than a score of 7) compared to others with a higher diversity score.

Factor	Level	High Diversity N = 262	Low Diversity N = 65	*p*-Value
Subtribe	Betta Kuruba	1 (0.4%)	0 (0.0%)	0.35
	Jenu kuruba	9 (3.4%)	5 (7.7%)	
	Kadu kuruba	4 (1.5%)	2 (3.1%)	
	Soliga	248 (94.7%)	58 (89.2%)	
Marital status	Married	260 (99.3%)	65 (100%)	0.19
	Widowed	2 (0.8%)	0 (0.0%)	
Occupation	Works in a shop but is not earning	262 (100.0%)	65 (100.0%)	
	Self-employed	262 (100.0%)	65 (100.0%)	
	Self-employed agriculture	262 (100.0%)	65 (100.0%)	
Taluk	Chamarajanagar	17 (6.5%)	21 (32.3%)	<0.001
	Gundlupet	38 (14.5%)	15 (23.1%)	
	Hanur	166 (63.4%)	6 (9.2%)	
	Kollegal	19 (7.3%)	2 (3.1%)	
	Yelandur	22 (8.4%)	21 (32.3%)	
Age (years), median (IQR)		24 (22, 28)	24 (23, 29)	0.54
Blood relationship with husband	Another blood relative	19 (7.3%)	20 (30.8%)	<0.001
	First cousin on my father's side	46 (17.6%)	4 (6.2%)	
	First cousin on my mother's side	47 (17.9%)	7 (10.8%)	
	Not a relative	86 (32.8%)	8 (12.3%)	
	Second cousin	62 (23.7%)	24 (36.9%)	
	Uncle	2 (0.8%)	2 (3.1%)	
BMI, median (IQR)		18.8 (17.11, 20.83)	19.3 (17.58, 22.01)	0.29
Education	0	31 (11.8%)	10 (15.4%)	0.44
	1	231 (88.2%)	55 (84.6%)	
Housewife	0	7 (2.7%)	0 (0.0%)	0.18
	1	255 (97.3%)	65 (100.0%)	
Marital status	Married	260 (99.3%)	65 (100%)	0.19
	Widowed	2 (0.8%)	0 (0.0%)	
MCV, median (IQR)		69.6 (64.8, 75.65)	71.25 (67.4, 77)	0.21
Electricity	0	44 (16.8%)	15 (23.1%)	0.24
	1	218 (83.2%)	50 (76.9%)	
Height (cm), median (IQR)		150.45 (147, 154.5)	150.6 (147.7, 153.7)	0.9
Weight (kg), median (IQR)		42.8 (38.4, 48)	44.6 (38.7, 49.8)	0.24
Gravida	1	94 (35.9%)	21 (32.3%)	0.83
	2	99 (37.8%)	28 (43.1%)	
	3	52 (19.8%)	10 (15.4%)	
	4	13 (5.0%)	5 (7.7%)	
	5	3 (1.1%)	1 (1.5%)	
	6	1 (0.4%)	0 (0.0%)	
Parity	1	107 (40.8%)	26 (40.0%)	0.53
	2	97 (37.0%)	27 (41.5%)	
	3	47 (17.9%)	7 (10.8%)	
	4	7 (2.7%)	4 (6.2%)	
	5	3 (1.1%)	1 (1.5%)	
	6	1 (0.4%)	0 (0.0%)	
Haemoglobin (g/dL), median (IQR)		11.5 (10.35, 12.7)	11.35 (10.5, 12.3)	0.33
Ferritin, median (IQR)		34.9 (16.33, 80.6)	28 (15.24, 51.57)	0.16
sTfR, median (IQR)		4.49 (3.36, 7.13)	5.23 (4.16, 8.36)	0.041
MCV, median (IQR)		69.6 (64.8, 75.65)	71.25 (67.4, 77)	0.21

**Note: IQR = Interquartile Range.**

While the multivariable linear regression indicates a statistically significant positive association between ferritin and haemoglobin levels (Coefficient = 0.007, 95% CI [0.003, 0.010], *p* < 0.001), it is important to note that ferritin is an acute-phase reactant that can be elevated in the presence of inflammation or infection. Given that CRP or other inflammatory markers were not included in the model, the observed association may be confounded by unmeasured inflammatory status. For instance, with a ferritin level of 56 ng/mL, it is unclear whether this reflects adequate iron stores or an inflammatory response. Adjusting for markers such as CRP would strengthen the validity of the findings and help clarify the true nature of the ferritin–haemoglobin relationship ( Table 4).

**Table 4.** Multivariable Linear Regression with Hb and sTfR as Outcomes and Diversity Score as a Predictor.

Predictor/Variable	Coef.	Std. Err.	t-value	*p*-value	95% Confidence Interval	Sig.
Hb as Outcome						
HDDD_Score	0.04	0.10	0.43	0.66	-0.16 to 0.25	
sTfR	-0.002	0.002	-1.27	0.20	-0.006 to 0.001	
Diversity (1 - Base 0)	0.03	0.38	0.10	0.92	-0.72 to 0.80	
Diversity 2	0.27	0.62	0.43	0.66	-0.95 to 1.49	
Diversity 3	0.30	0.89	0.34	0.73	-1.45 to 2.05	
Ferritin	0.007	0.002	3.86	0.00	0.003 to 0.01	***
Age	-0.01	0.02	-0.38	0.70	-0.067 to 0.04	
Education	0.06	0.04	1.45	0.14	-0.02 to 0.15	
BMI	0.06	0.03	1.98	0.04	0.000 to 0.13	**
Socioeconomic Status	0.84	0.49	1.71	0.08	-0.12 to 1.82	*
Constant	8.95	1.69	5.27	0.00	5.60 to 12.30	***
sTfR as Outcome						
HHFD_Score	-2.97	1.47	-2.02	0.05	-5.88 to -0.00	**
Age	-1.24	1.22	-1.02	0.30	-3.66 to 1.166	
Parity	7.92	5.91	1.34	0.18	-3.73 to 19.58	
Education	-2.54	1.36	-1.87	0.06	-5.23 to -0.14	*
BMI	-4432.47	13553.88	-0.33	0.74	-31150.11 to 22285.16	
Constant	78.77	41.04	1.92	0.05	-2.12 to 159.68	*

Note: *** p < 0.01, ** p < 0.05, * p < 0.1. The model is adjusted for confounders, including age, education, BMI, socioeconomic status, dietary intake, nutritional markers, etc.

Multiple potential confounders are included in the Methods section. However, in the final multivariable regression model ( Table 3), the authors adjusted for a subset of these variables based on the DAG approach, ensuring that only the most relevant confounders were retained to minimize bias while avoiding overadjustment. This selection aligns with the analytical framework and the theoretical considerations guiding the study. The multivariable linear regression analysis shows that HHDD_Score negatively associates with sTfR, the dependent variable ( *β* = −2.975, *p* = 0.045), indicating that higher HHDD_Score values were associated with lower sTfR. Education also exhibited a negative association ( *β* = −2.547). Other predictors were not statistically significant ( *p* > 0.1) ( Table 4).

In the statistical analysis, sTfR was treated as a continuous variable rather than being categorized into levels. The logistic regression model assessed the association between sTfR and anaemia as a binary outcome (Hb < 10 g/dL), ( Table 5) with the odds ratio reflecting the increased likelihood of anaemia for each unit increase in sTfR. This should be explicitly mentioned in the Statistical Analysis section to clarify how sTfR was handled in the model.

**Table 5.** Multivariable Logistic Regression for Anaemia (Hb <10 g/dL) with sTfR as Predictor.

Variable	OR	*p*-value	95% Confidence Interval	Significance
sTfR	1.68	0.000	(1.40, 2.01)	***
Education (Reference: No Education)				
— Primary Education	1.006	0.99	(0.22, 4.53)	
— Secondary Education	1.33	0.73	(0.26, 6.75)	
— Higher Education	0.73	0.70	(0.14, 3.76)	
Ferritin	0.99	0.40	(0.97, 1.00)	
Age	0.96	0.62	(0.80, 1.11)	
BMI	0.93	0.38	(0.79, 1.09)	
Education (Continuous)	1.02	0.84	(0.82, 1.27)	
Constant	0.04	0.23	(0.00, 7.05)	

Significance levels: *** p < 0.01, ** p < 0.05, * p < 0.1.

Although the ferritin level of 56 ng/mL is within the normal range and might suggest adequate iron stores, logistic regression results showed a significant association between sTfR and anaemia (Hb < 10 g/dL)—with an odds ratio of 1.68 (95% CI: 1.404–2.019, *p* < 0.01)—indicating that sTfR is capturing functional iron deficiency or increased erythropoietic activity not reflected by ferritin alone. sTfR levels rise in response to cellular iron demand and tissue iron insufficiency, which can occur even when ferritin is normal, particularly in conditions of chronic disease or inflammation. Therefore, the strong association likely reflects a more sensitive detection of iron-restricted erythropoiesis, supporting the utility of sTfR as a complementary marker in identifying early or functional iron deficiency contributing to anaemia. Other covariates, including age, education, BMI, and ferritin, did not show a statistically significant association with anaemia, as reflected by their non-significant *p*-values ( *p* > 0.05) and odds ratios close to 1.

**Figure 3.**
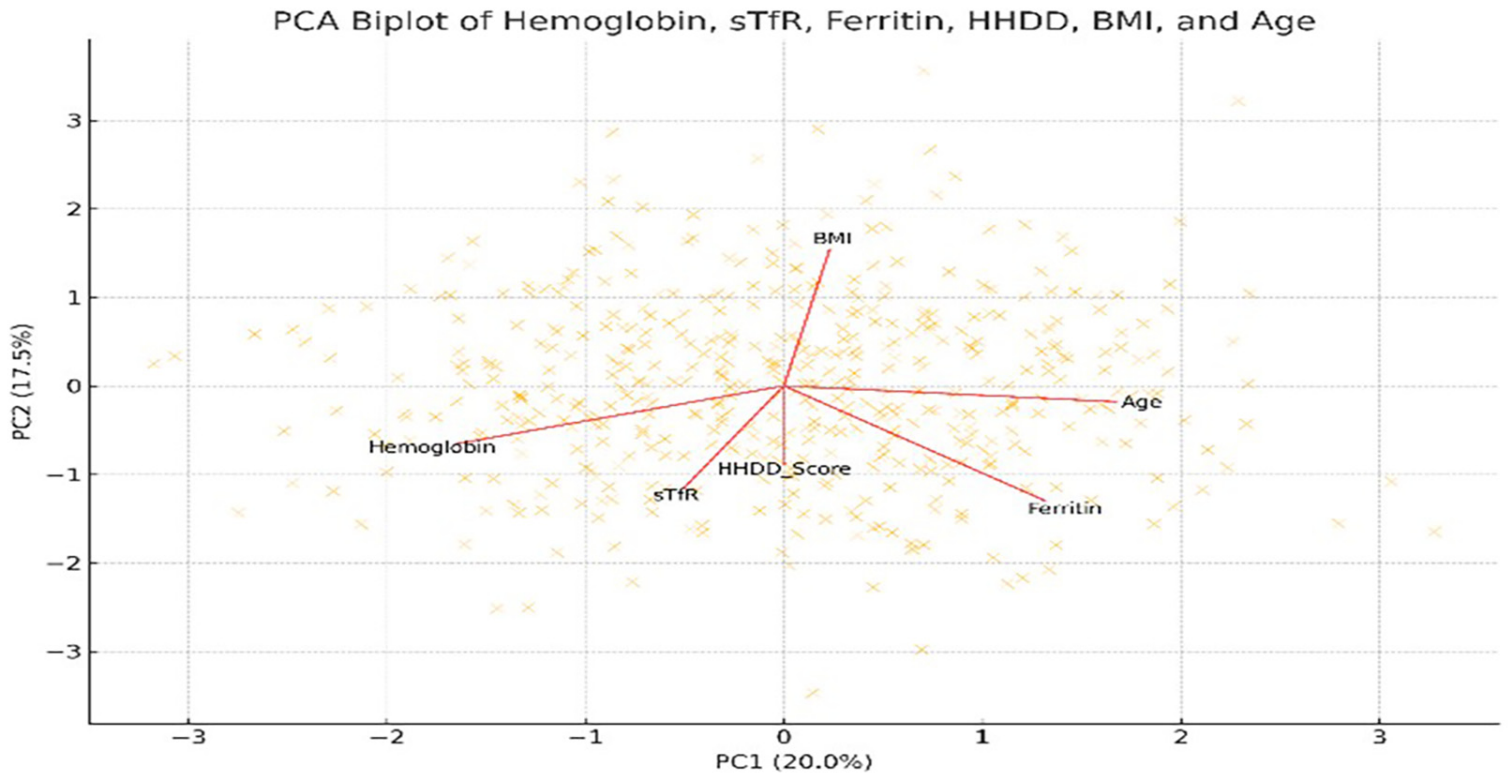
Principal Component Analysis (PCA) biplot of dietary, biochemical, and demographic variables among Adivasi women in the chiguru cohort.

The biplot illustrates ( Figure 3) the relationships among haemoglobin, ferritin, soluble transferrin receptor (sTfR), household dietary diversity score (HHDD), body mass index (BMI), and age. Arrows represent variable loadings on the first two principal components. Opposing directions of HHDD and sTfR reflect their inverse relationship observed in regression analysis. Haemoglobin and ferritin cluster closely, indicating shared patterns in iron status markers.

## Discussion

The study reveals a complex relationship between dietary diversity, iron metabolism, and anaemia risk among Adivasi women. While HHDD scores did not directly predict haemoglobin levels, they were inversely associated with sTfR levels, indicating that increased dietary diversity may enhance iron availability and metabolism.

In many remote Indigenous communities, anaemia screening traditionally relied solely on haemoglobin measurements due to limited laboratory facilities and indicators influenced by factors beyond iron status ^27^. Reliance on this measure alone obscures the underlying aetiology of anaemia. The authors show that using serum ferritin and sTfR increases the prediction of iron deficiency in vulnerable areas. These biomarkers are used in tribal communities where infections and malnutrition often coexist, distinguishing iron-deficiency anaemia from chronic disease.

Dietary diversity is essential for iron availability and anaemia risk in resource-poor populations. Monotonous staple-based diets, high in phytates and low in bioavailable iron, contribute to high anaemia prevalence. A diverse diet, along with animal-source foods and vitamin C, fruits, and vegetables, increases iron absorption. Consuming ascorbic acid counteracts the inhibitory effects of phytates and polyphenols, resulting in better iron absorption and utilization. Consistent with biological mechanisms, the findings show a positive association between greater dietary diversity and improved iron status, as indicated by lower sTfR concentrations—a sensitive marker of functional iron deficiency—despite the absence of ferritin, which reflects iron stores. Prior studies suggest that sTfR provides reliable insight into tissue iron demand, especially in inflammation-prone populations where ferritin may be elevated independently of iron status ^28^, which demonstrated that improved dietary intake reduces sTfR levels by enhancing iron absorption and utilization ^29^. While lower dietary diversity has been associated with elevated sTfR levels, indicating suboptimal iron status, it is also important to consider that increased iron demands during pregnancy may independently raise sTfR concentrations regardless of intake ^30^. Individuals consuming a wider variety of food groups showed higher haemoglobin concentrations and more favourable iron biomarker profiles, indicating more robust iron stores and transport capacity. This observation aligns with evidence from other regions. In a study of school-aged children in South Africa, low dietary diversity (≤4 out of 9 food groups) was associated with significantly higher odds of anaemia and iron deficiency. In contrast, diets including fruits, vegetables, and animal-source foods were protective ^31^. Similarly, pregnant women in Cameroon who achieved a more diverse diet had markedly higher mean haemoglobin levels and a lower prevalence of anaemia compared to those with less diverse diets. Conversely, where dietary diversity is minimal, iron deficiency remains pervasive: for instance, the Indigenous population of Jharkhand showed an inverse relationship between minimum dietary diversity and the biomarker sTfR, suggesting a reduction in iron deficiency biomarkers, including ferritin. The cross-sectional analysis of a cohort of singleton pregnant women demonstrated that dietary diversity did not exhibit a statistically significant association with iron deficiency anaemia, as determined by serum ferritin levels. The observed decline in sTfR with increasing dietary diversity highlights the importance of diversified diets in improving iron metabolism. sTfR is an emerging biomarker of iron status that reflects tissue-level iron demand and erythropoietic activity more accurately than ferritin, which is influenced by inflammation. Hence, improving diet diversity, particularly by incorporating iron-rich foods (meat, legumes, leafy greens) and nutrient-dense traditional foods, can substantially improve iron biomarkers and reduce anaemia risk in vulnerable populations ^32^. The concordance between the results and these comparative studies strengthens the evidence that dietary diversity is a key leverage point for enhancing iron metabolism in settings of endemic malnutrition.

The findings carry critical public health relevance, especially for Indigenous communities facing a disproportionate anaemia burden. With a mean haemoglobin level of 11.42 g/dL—below the WHO threshold for normality in women and children—this population likely experiences anaemia of moderate public health significance ^33^. Moderate dietary diversity observed in the cohort likely constrained micronutrient intake, particularly iron, folate, and vitamin A, which are essential for haemoglobin synthesis ^34^. Studies suggest that improving dietary diversity from moderate to good could significantly raise haemoglobin levels and reduce anaemia prevalence by up to 20–30% ^35^. Addressing anaemia in these populations requires a multifaceted approach: enhancing food diversity through culturally relevant agricultural programs and nutrition education, scaling up IFA supplementation, and strengthening health systems for routine anaemia screening and treatment ^36^. Integrating Indigenous health workers and traditional knowledge may further improve acceptability and impact ^37^.

A diet comprising diverse food groups—such as cereals, legumes, green leafy vegetables, fruits, and animal-source foods—supplies a broad spectrum of essential nutrients, notably iron and compounds such as vitamin C that improve iron absorption. In Adivasi populations, where reduced access to forest-based foods and existing nutritional deficiencies are prevalent, iron deficiency is a major concern. Therefore, enhancing dietary diversity is a key strategy to improve iron levels within these communities. The authors observed a strong link between elevated sTfR, indicating iron deficiency, and anaemia. The odds of anaemia increased by approximately 68.4% for each unit increase in sTfR, highlighting the critical role of iron deficiency as reflected by high sTfR in the population. The findings support earlier research in Jharkhand’s Indigenous communities, which also showed a strong link between folate-rich foods and improved iron status markers like ferritin, sTfR, and haemoglobin ^1^. Similar findings have been observed in research on anaemia in inflammatory and chronic disease conditions, where sTfR better distinguishes IDA from ACD ^38^. An Indian study showed that iron supplements lowered sTfR levels in pregnant women with iron deficiency anaemia, confirming better iron status ^38^. A study evaluating sTfR diagnostic accuracy for iron deficiency anaemia versus beta-thalassemia found that similar results have been obtained from another country, along with similar other results, which have shown a positive correlation between the nutritional biomarker and iron deficiency status ^39, 40^.

As observed in the study, it aligns with previous research indicating that food security and local agricultural practices significantly impact anaemia prevalence ^41^. Addressing anaemia in these populations requires targeted interventions that incorporate both dietary and non-dietary factors, including infection control, sanitation improvements, and culturally appropriate supplementation programs ^42^.

Strengthening traditional food systems—defined as locally rooted agricultural, foraging, and food preparation practices passed down through generations—increases the availability of iron- and vitamin-rich foods and ensures alignment with local cultural preferences, ecological knowledge, and seasonal food access. Furthermore, integrating the assessment of new iron biomarkers into public health programs can enable more effective monitoring and targeting. In community health screenings, routine measurement of indicators like ferritin, sTfR, and C-reactive protein would help distinguish true iron deficiency from inflammation-driven anaemia, ensuring that interventions (dietary diversification, fortification, or therapy for infections) are appropriately tailored. In summary, a multifaceted approach that combines improved dietary diversity, utilization of Indigenous foods, and biomarker-informed surveillance could significantly advance anaemia prevention in Indigenous settings. The findings have significant implications for public health interventions aimed at reducing anaemia prevalence. Improved dietary diversity could be an effective approach to address iron deficiency and reduce anaemia prevalence at the population level. The Adivasi population experiences a disproportionate burden of anaemia due to the synergistic effects of infectious and inflammatory processes stemming from inadequate sanitary conditions and open defecation, coupled with suboptimal dietary intake and nutrient deficiencies, which collectively impair iron availability and absorption. To better understand and address anaemia, future studies should investigate anaemia profiles in men and children, identify contributing factors, and inform corrective strategies.

The observed discrepancy, wherein dietary diversity exhibited no significant association with haemoglobin levels but demonstrated a correlation with sTfR, may be attributed to the differential sensitivity of these markers. Specifically, haemoglobin reflects the concentration of red blood cells, whereas sTfR serves as a more sensitive indicator of iron deficiency at the cellular level. So, the association between dietary diversity and sTfR is logical from a biological perspective.

The findings reconfirm the importance of Adivasi food sources and their contribution to nutritional status. Strategies should focus on increasing awareness of access to nutrient-rich foods, promoting the production and consumption of balanced diets, and facilitating early anaemia testing while addressing underlying infections. The efficacy of homestead food production initiatives, coupled with nutrition education, has been demonstrated across Bangladesh, Cambodia, Nepal, and the Philippines, with documented improvements in food diversity and reductions in the prevalence of anaemia ^43^.

The cross-sectional nature of the study design precludes the establishment of causal relationships, limiting the findings to observed associations. Furthermore, the potential for recall bias in the dietary recall data and the reliance on household-level food access information, which may not accurately represent individual women’s consumption, represent methodological limitations. These findings highlight the need to integrate sTfR measurements into anaemia screening protocols, as conventional markers like ferritin may be confounded by inflammation. The inclusion of sTfR could enhance the accuracy of iron deficiency diagnoses and facilitate targeted nutritional interventions.

## Strengths of the Study

This study offers several strengths. First, it uniquely combines biochemical iron biomarkers (sTfR and ferritin) with dietary diversity data (HHDD scores), enabling a multidimensional assessment of iron status among Adivasi women—a population underrepresented in the existing literature. Second, the inclusion of sTfR, a markerless effect of inflammation, improves diagnostic specificity for iron deficiency. Third, the study is embedded within an ongoing community cohort (chiguru), ensuring rich contextual data and sustained community engagement. Using culturally appropriate tools and involving Adivasi staff improved the quality of data and made the research more acceptable to the community, following participatory and decolonized research practices.

## Conclusions

This study highlights the critical role of dietary diversity in influencing iron metabolism and the importance of sTfR as a key biomarker for anaemia risk assessment among Adivasi women. While dietary diversity was not directly predictive of haemoglobin levels, its inverse association with sTfR suggests that improving dietary variety can enhance iron status and reduce iron demand at the tissue level. Furthermore, sTfR emerged as a strong predictor of anaemia, reinforcing its potential as a diagnostic tool for targeted interventions in resource-poor settings.

The findings emphasize the need for culturally relevant strategies to combat anaemia in tribal communities. Promoting the cultivation and consumption of locally available iron-rich foods and encouraging community-led agricultural initiatives can significantly improve dietary diversity. For example, leafy greens such as amaranthus (dantina soppu), drumstick leaves (moringa), colocasia leaves (kesuvina soppu), and wild legumes traditionally gathered or cultivated by Adivasi communities are rich in iron and commonly consumed. Strengthening support for kitchen gardens, seasonal forest-based harvesting, and community seed banks can further empower these populations to preserve their dietary heritage while improving nutritional outcomes. Additionally, strengthening public health interventions by integrating sTfR-based screening and expanding healthcare infrastructure in tribal regions will be essential for early anaemia detection and effective management.

In conclusion, a multidimensional approach that combines nutritional interventions, improved screening protocols, and community engagement is essential for sustainable anaemia reduction. Addressing the underlying barriers will be key to making lasting improvements in nutrition outcomes and overall well-being among Adivasi women. Further research should explore the causes of anaemia and factors driving it across the population level, including men and children, to develop more precise and effective intervention strategies.

Funding: This study was funded by the DBT/Wellcome Trust India Alliance Clinical and Public Health Research Centers grant for the Centre for Training, Research, and Innovation in Tribal Health (CTRITH) [IA/CRC/20/1/600007] awarded to Prashanth NS, Suresh Shapeti, Deepa Bhat, and Upendra Bhojani (support to MK, PS, SS, and TS).

## Institutional Review Board Statement

The Institutional Ethics Committee (IEC) reviewed and approved the study at the Bangalore campus of IIPH-H (Approval number IIPHHB-TRCIEC-216-2021, dated May 6, 2024). Only participants who are willing to participate voluntarily and those who have provided written informed consent are enrolled.

## Informed Consent Statement

Written informed consent was obtained from all subjects involved in the study. Only women aged 18 years and above were enrolled; no minors participated. For illiterate participants, the consent process included an oral explanation in the local language and witnessed thumbprint consent.

## Abbreviations

The following abbreviations are used in this manuscript:

**Table:** 

HHDD	Household Dietary Diversity
sTfR	Soluble Transferrin Receptor
WHO	World Health Organization
CRP	C-Reactive Protein
AGP	Alpha-1-Acid Glycoprotein
IDA	Iron Deficiency Anaemia
NFHS	National Family Health Survey
FFQ	Food Frequency Questionnaire
FAO	Food and Agriculture Organization
ROC	Receiver Operating Characteristic
AUC	Area Under the Curve
VGKK	Vivekananda Girijana Kalyana Kendra
Hb	Haemoglobin
MCV	Mean Corpuscular Volume
RBC	Red Blood Cell
DAG	Directed Acyclic Graph
BMI	Body Mass Index
IFA	Iron–Folic Acid
ACD	Anaemia of Chronic Disease

## Data Availability

The datasets generated and/or analyzed during the current study are not publicly available due to ethical restrictions and protection of participant confidentiality, as required by the Institutional Ethical Review Board of the Indian Institute of Public Health–Bangalore (Approval number IIPHHB-TRCIEC-216-2021, dated 6 May 2024). The IRB specifically mandated that individual-level data containing sensitive health and demographic information from Adivasi women should not be placed in an open repository to prevent risks of misuse or breach of privacy. Data access can be granted upon reasonable request to the corresponding author ( maithili.k@phfi.org) under a data sharing agreement. Access will only be provided to qualified researchers for non-commercial academic purposes, after approval by the Institutional Review Board, and under conditions that ensure data security and participant confidentiality. Extended materials supporting the study (survey tools and summary files) are openly available via Zenodo at: https://doi.org/10.5281/zenodo.17301153 (V.01) Zenodo. *Dietary Diversity, Iron Status, and Anaemia Among Adivasi Women: Insights from the chiguru Cohort in Chamarajanagar District, Southern Karnataka*. https://doi.org/10.5281/zenodo.17301153 (V1.0) ^44^ This project contains the following underlying data: CTRITH-Cohort-Baseline Questionner-Final_26.04.2022.docx Field_Data_Collection_Checklist (1).pdf Figure 1.jpg Figure 2.jpg Figure 3.jpg PIS-consent from_English.docx PIS-consent_final_kannada.docx Qunatitative-data-sheet.xlsx Data is available under the terms of the Creative Commons Attribution 4.0 International (CC BY 4.0) license. Zenodo repository DOI : https://doi.org/10.5281/zenodo.17301153 (V1.0)
