# Dietary Diversity, Iron Status, and Anaemia Among Adivasi Women: Insights from the Chiguru Cohort in Chamarajanagar District, Southern Karnataka

**DOI:** 10.12688/wellcomeopenres.24846.2

**Published:** 2026-02-02

**Authors:** Maithili Karthik, Jai Prabhakar Sosale Chandrashekaraswamy, Prafulla Shriyan, Suresh Shapeti, Prashanth Thankachan, Tanya Seshadri, Giridhara R Babu

**Affiliations:** 1 Public Health, Indian Institute of Public Health, Bengaluru, Karnataka, 560038, India; 2 Anthropology, Karnatak University Dharwad, Dharwad, Karnataka, 580003, India; 3 Anthropology, Centre for Multi-disciplinary Development Research (CMDR), Dharwad, Karnataka, 580004, India; 4 Division of Nutrition, St. John’s Research Institute, Bengaluru, Karnataka, 560034, India; 5 Centre of Adivasi Health, Institute of Public Health Bengaluru, Bengaluru, Karnataka, 560070, India; 6 Population Medicine, College of Medicine, QU Health, Qatar University College of Medicine, Doha, Doha, Qatar

**Keywords:** anaemia; dietary diversity; soluble transferrin receptor; adivasi women; iron deficiency; tribal health

## Abstract

**Introduction:**

Anaemia remains a significant public health concern, particularly among marginalized populations such as tribal communities. This study examines the complex relationships between dietary diversity, iron status biomarkers, and anaemia prevalence among Adivasi women in southern Karnataka, India.

**Method:**

A cross-sectional analysis using data from the Chiguru Adivasi cohort in Chamarajanagar District, Karnataka, India. The study included 479 women. PNC women aged 18 and above, and other women in the family aged 18–65 years, primarily from the Soliga tribe. The authors assessed Household Dietary Diversity Score (HDDS)—a measure of food variety—and measured hemoglobin (Hb), soluble transferrin receptor (sTfR) levels, and ferritin, along with sociodemographic data.

**Results:**

The HDDS did not directly predict Hb levels but was negatively associated with sTfR (β = –2.975, p = 0.045). After adjusting for confounders, sTfR was a significant predictor of anaemia (OR = 1.68, 95% CI: 1.40–2.01, p < 0.01). Geographic variations in dietary diversity among the Soliga community, assessed through stratified analysis of Household Dietary Diversity Scores (HDDS) across taluks and proximity to forested areas, were found to be influenced by forest access, seasonal availability of wild foods, and local agricultural practices.

**Conclusions:**

This study highlights the role of dietary diversity and iron biomarkers in understanding anaemia among Adivasi women. While household dietary diversity was not directly associated with hemoglobin, it showed a significant inverse relationship with soluble transferrin receptor (sTfR) levels, indicating improved tissue-level iron status with more diverse diets. Elevated sTfR levels were strong predictors of anaemia, reinforcing their utility as sensitive biomarkers for early iron deficiency. These findings emphasize the need for culturally relevant, cost-effective interventions that promote locally available iron-rich foods and strengthen community-led nutrition programs to enhance both dietary quality and iron status. Integrating these biomarker-informed insights into public health strategies can contribute to more targeted and sustainable anaemia reduction in tribal populations.

## Introduction

Anaemia is a major global health issue, disproportionately affecting tribal communities, which in the Indian context refer to constitutionally recognized Scheduled Tribes (Adivasi groups) who are Indigenous populations characterized by distinct cultural, linguistic, and socio-economic identities, often residing in geographically isolated forested or hilly regions. Globally, an estimated 1.62 billion people—about one in four individuals—are affected by anaemia, with the highest prevalence among women and children ^1, 2^. In India, anaemia affects 57% of women of reproductive age, reflecting a major public health challenge ^3^. Among India’s tribal populations, the burden is substantially higher, with prevalence ranging from 68% to over 90% across regions ^4, 5^. The present study focuses on Adivasi (Indigenous) women from the Soliga, Jenu Kuruba, and Kadu Kuruba communities of southern Karnataka, who represent some of the most socio-economically and geographically marginalised groups. Anaemia in pregnancy is associated with severe health implications, including impaired cognitive and physical performance, increased morbidity, and adverse maternal and child health outcomes ^6, 7^.

Dietary diversity is a key determinant of nutritional adequacy and is particularly relevant in tribal populations, where diets often consist of staple foods with limited variety ^8^. Consuming a diverse diet rich in iron-containing foods and other essential micronutrients is associated with better health outcomes, including reduced risk of anaemia ^9^. However, evidence linking dietary diversity to anaemia among tribal populations is sparse. Studies have shown that tribal communities often have diets dominated by cereals and starchy staples ^8, 10, 11^. In these settings, daily meals typically consist of rice or millets as the main staple, accompanied by small quantities of pulses and seasonal vegetables. Consumption of animal-source foods is occasional, while fruits and dairy products are consumed infrequently. Low consumption of dairy products, fruits, and vegetables leads to inadequate intake of key nutrients like iron, calcium, and vitamin A ^12^. Protein intake can also influence iron absorption and metabolism; for example, proteins from legumes and soy may inhibit iron absorption because of their high phytate content ^13^. This limited dietary diversity contributes to high rates of anaemia and other micronutrient deficiencies in these populations.

Despite being an acute-phase reactant, ferritin is currently the best available biomarker for assessing iron stores, as recommended by the World Health Organization WHO. While its levels can be influenced by inflammation and infection, adjustments based on C-reactive protein CRP and alpha-1-acid glycoprotein AGP are ideal but were not feasible in the present study due to logistical limitations. The potential influence of hemodilution in pregnancy and obesity is acknowledged as a confounder, though the interpretation of iron status has been made cautiously within this context ^14^. In contrast, soluble transferrin receptor sTfR is a reliable marker of tissue iron deficiency, which rises earlier in pregnancy and remains unaffected by inflammation or infection. The sTfR is a sensitive indicator of iron status and iron-deficiency anaemia ^15^, reflects tissue-level iron demand and may be an early marker of iron deficiency, even in individuals with normal systemic iron parameters. The sTfR index, calculated as the ratio of sTfR to the logarithm of ferritin, has been shown to improve the detection of iron deficiency anaemia compared to other markers ^16, 17^.

While iron biomarkers are helpful, they are only part of the picture of anaemia, particularly in marginalized or tribal populations, where there is a plethora of nutritional, infectious, and environmental factors. Field research has demonstrated that food diversity, as a measure of micronutrients alongside iron intake, is a better predictor of anaemia than single-nutrient biomarkers in isolation ^3, 18^. Most existing studies focus either on dietary patterns or biochemical markers in isolation, failing to capture their interplay. This gap is particularly pronounced in tribal populations, where unique cultural, dietary, and environmental factors may influence anaemia prevalence differently than in general populations. For instance, tribal communities often face limited access to nutritious foods, including iron, folate, and vitamin B12, which can contribute to higher anaemia rates ^19, 20^. Additionally, cultural norms, poor sanitation practices, and insufficient maternal care may collectively impact anaemia prevalence among women of reproductive age in these communities ^4^. Addressing this research gap is crucial for designing targeted interventions that are culturally and contextually appropriate, as the prevalence of anaemia in some tribal populations has been reported to be as high as 89–96.5% ^5^. This study combines dietary diversity assessment with iron biomarker analysis, the authors aim to understand the aetiology of anaemia in tribal populations ^21^. By exploring the relationship between nutritional diversity and iron biomarkers, the research aims to identify significant predictors of anaemia and deepen the understanding of anaemia in tribal populations. This can inform the design of targeted interventions that consider these populations’ distinctive needs and contexts, potentially leading to more effective anaemia prevention and control in vulnerable populations. We hypothesize that increased household dietary diversity score (HDDS) is associated with improved iron status, measured as decreased soluble transferrin receptor (sTfR) levels and decreased risk of iron deficiency anaemia (IDA) in Adivasi women. The association remains strong even after adjusting for the key sociodemographic and nutritional confounding factors.

## Objective

We examined the association between dietary diversity and iron deficiency anaemia (IDA) using soluble transferrin receptor (sTfR) levels and investigated potential risk factors for IDA in the tribal cohort.

## Methodology

### Study setting

**Figure 1.**
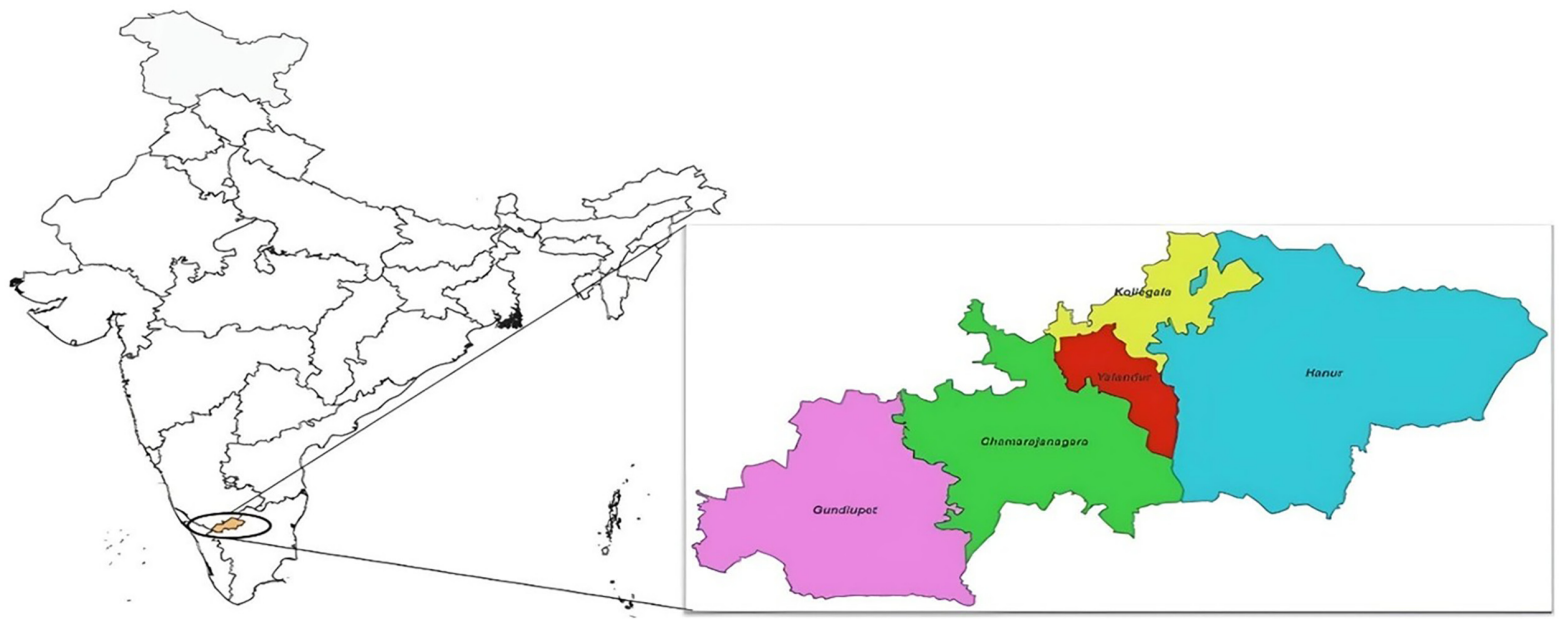
Chamrajanagar district study area.

**Figure 2.**
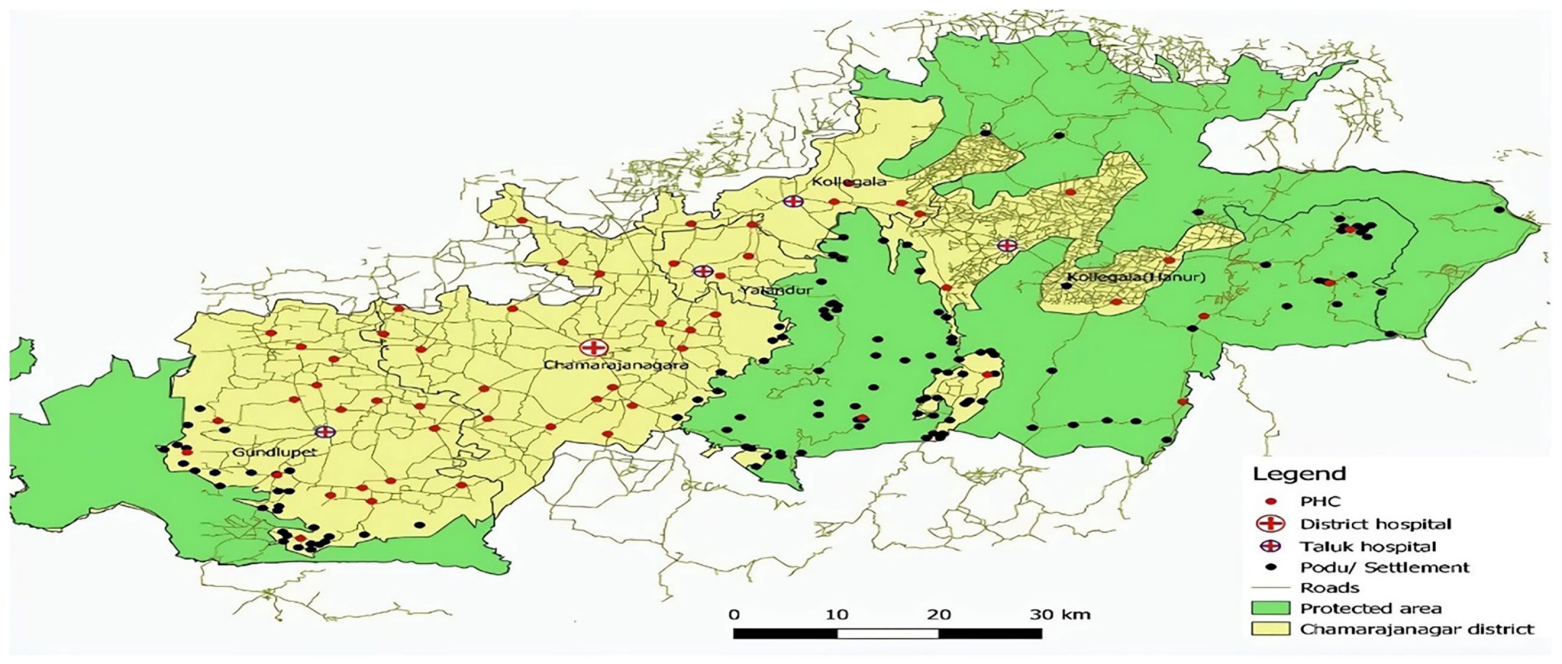
The black dots represent the settlements of both Adivasi communities within and around the protected areas (Green).

This is a cross-sectional study of Adivasi women (Indigenous tribal community women) conducted in the Chiguru Adivasi family cohort living in the Chamrajanagar district of Southern Karnataka ^10^. As per National Family Health Survey – 5 NFHS-5, the prevalence of anaemia is 46.3% in Chamrajanagar District. We used a standard sample size formula for estimating anaemia prevalence (46.3%) at a 95% confidence level. The final sample of 479 provided approximately 80% power, ensuring a reliable estimation of anaemia prevalence within the given confidence interval. The study was conducted in Chamrajanagar District, Karnataka, located between Tamil Nadu and Kerala in the Nilgiris region ( Figure 1). Approximately 48% of its total land area is covered by forests, with substantial portions designated as regions protected under the Wildlife Protection Act of 1972. About 48% of the district is forested, including Bandipur, Biligiriranga Hills, and Malai Mahadeshwara wildlife sanctuaries. The main tribal groups in this region are Soliga, Jenu Kuruba, and Kadu Kuruba, who live in forest settlements called *Podus*. These communities have established unique habitats within this region, contributing to the diverse cultural landscape. The Podus are unique settlement clusters that serve as the primary living spaces for different Soliga tribal groups. The Soligas, a Scheduled Tribe concentrated in and around the Biligiri Rangaswamy Temple Tiger Reserve, primarily depend on farming, forest products, and wage labour. They speak the Soliga (Dravidian) language and have limited access to health and education services. Each *Podu* consists of 20–200 people and serves as a self-sufficient community unit ^11^. The majority of the families are of low socioeconomic status and have limited health and education services ^22^. ( Figure 2) The black dots represent the settlements of both Adivasi communities within and around the protected areas (Green). Each Podu functions as a self-sufficient unit, fostering a strong sense of community, cultural identity, and traditional practices. These settlements are typically located in forested regions, reflecting the Soligas’ deep connection with nature and their sustainable way of life. The Podu system plays a crucial role in preserving their heritage, social structure, and Indigenous knowledge across generations of Chamrajanagar. The geographical setting and cultural diversity of these tribal communities contribute to the area’s unique identity and heritage.

### Inclusion and exclusion criteria

The research targeted participants who live in and around Chamrajanagar and the surrounding talukas (administrative sub-districts), focusing on tribal populations like the Soliga, Jenu Kuruba, and Kadu Kuruba. The inclusion criteria are postnatal women and their female relatives over 18 years of age who are willing to consent to baseline data collection, anthropometric measurements, and blood sampling. Postnatal women were recruited as part of the Chiguru Adivasi family cohort and were followed up at 3 months, 6 months, and 1 year postpartum. At the time of recruitment, all eligible female family members aged 18 years and above residing in the same household were also approached for participation. They are also enrolled in the chiguru Adivasi family cohort. Exclusion criteria encompass non-tribal individuals who do not consent to participate will be excluded from the research.

### Informed consent

Only women aged 18 years and above were enrolled in this study; no minors participated. There was no predefined upper age limit for participation, and all eligible adult women within the household, irrespective of age, were invited to take part. Written informed consent was obtained from all participants before data collection. For illiterate participants, the consent process was explained orally in the local language, and their consent was documented using a thumbprint in the presence of a literate witness. This approach was used instead of written consent to ensure inclusivity and ethical participation of individuals unable to read or write. This study was conducted and reported in accordance with the Strengthening the Reporting of Observational Studies in Epidemiology (STROBE) guidelines to ensure transparency and completeness in reporting.

### Data collection

The HDDS Score is the primary exposure variable in this study, Dietary diversity is particularly significant in tribal populations due to the influence of traditional food habits, seasonal availability, and socioeconomic factors. Data collection for the cohort was conducted between May 2023 and December 2024, capturing dietary information during both the monsoon and post-harvest seasons. To examine geographic variations in dietary diversity, the HDDS scores were compared across the five taluks (Hanur, Gundlupet, Chamarajanagar, Kollegal, and Yelandur), representing different levels of forest proximity and agricultural dependence. This stratification enabled the identification of spatial patterns related to forest access and seasonal food availability. Additionally, food availability in these communities varies seasonally, with forest-based foods such as green leafy vegetables, tubers, and wild fruits being more abundant during the monsoon, and dependence on purchased staples increasing during the dry months. Therefore, while the data span multiple periods of food access, certain micro-seasonal variations in diet composition may not have been fully represented. It is derived from a validated Food Frequency Questionnaire (FFQ) that categorizes food groups based on international standards, such as the Food and Agriculture Organization (FAO) FAO and HDDS classifications. Participants report their consumption of these food groups within a specified recall period (e.g., 24 h to 7 days) ^23^. The HDDS is calculated by summing the number of food groups consumed, with higher scores indicating better dietary diversity and nutrition, while lower scores reflect food insecurity and poor diet quality. Each food group consumed was assigned a score of 1, while non-consumption was scored as 0, resulting in a possible HDDS range of 0–12. For analytical purposes, HDDS scores were categorised into low, medium, and high dietary diversity. The aggregation of food groups used to construct the HDDS is presented in Supplementary Table S1.

The (HDDS) used in this study was derived from the FAO (HDDS) guidelines. The HDDS ranges from 0–12, indicating the total number of food groups consumed by the household in the last fifteen days (1 = consumed, 0 = not consumed). Dietary information was collected from women who serve as reliable reporters of household food access and meal preparation. Thus, the HDDS reflects household-level diversity rather than individual intake. While conceptually similar to the Minimum Dietary Diversity for Women (MDD-W), which is used for individual dietary adequacy, only the HDDS framework was applied in this study to capture household food diversity in the tribal context.

Due to the influence of traditional food habits, seasonal availability, and socioeconomic factors. HDDS provides a quantitative measure of dietary practices and their potential link to anaemia. It was collected from postnatal mothers through a self-reported questionnaire at the study’s initiation phase to capture early postpartum dietary patterns. HDDS has demonstrated strong internal consistency and repeatability ^24^. The Household Dietary Diversity Score (HDDS) has shown internal consistency and has been successfully applied across many different cultures, even in the case of tribal societies with diversified and localized food systems. The high inter-rater reliability in studies conducted in India ^23^. Additionally, the HDDS has been validated by the FAO as a predictor of food security, with an area under the ROC curve AUC of 0.89, indicating high predictive accuracy ^25^. Additionally, its reliability across recall periods has been confirmed, with no significant differences found between 24-h and 7-day recall periods ( *p* > 0.05) ^26^.

Iron status was assessed using soluble transferrin receptor (sTfR) and ferritin, with sTfR reflecting tissue-level iron deficiency and ferritin representing body iron stores. Which has a dual role in reflecting iron metabolism and, as a predictor variable, specifically targets iron deficiency anaemia rather than anaemia of other etiologies. Phlebotomists prepared and collected blood samples using standardized processes. For PNC women, we did not collect information on their menstrual cycle. For other adult women in the household, menstrual cycle details at the time of blood collection were also not recorded. With informed consent, biological samples were obtained from individuals at the PODUs by trained phlebotomists at B.R. Hills. The samples were processed at the VGKK laboratory, subsequently transported to the Vailes biorepository, sealed with Parafilm, and cryo-labels preserved at −80 °C for later analysis of sTfR and ferritin levels.to maintain their integrity. Temperature checks were conducted, and meticulous sample recording was done in the logbook. All blood samples and dietary diversity questionnaires were collected at baseline during the same data collection visit, ensuring that biochemical and dietary assessments were temporally aligned.

### Laboratory analysis

Hemoglobin (Hb), mean corpuscular volume (MCV), and red blood cell (RBC) count were measured using the Sysmex XN-1000 automated haematology analyser (Sysmex Corporation, Kobe, Japan). Serum ferritin was analysed using the Chemiluminescent Microparticle Immunoassay (CMIA) kit (Abbott Diagnostics, Cat# 7K60-20, Abbott Laboratories, USA). Soluble transferrin receptor (sTfR) was measured using the Human sTfR ELISA kit (R&D Systems, Minneapolis, USA; Cat# DTR100). Quality control was performed using Randox QC materials (Randox Laboratories, Crumlin, UK; Cat# HE1532). All assays and reagents were used according to the manufacturer’s instructions. Serum ferritin and sTfR measurements were done at baseline, during the first round of data collection, and was not assessed among other adult female household members.

Using a DAG ( Figure 4), a Directed Acyclic Graph (DAG) was used to identify potential confounders and guide variable selection for multivariable analysis based on prior evidence and conceptual relationships. The DAG was used to identify the minimal sufficient adjustment set to control for confounding while avoiding overadjustment by excluding mediators on the causal pathway. The authors adjusted for sociodemographic factors (age, marital status, tribe or subtribe, household size, and education), socioeconomic and household factors (SLI, occupation, water source, and sanitation), parity, infection history (malaria or parasitic infection), BMI, and other relevant anthropometric measures. As shown in Figure 4, the hypothesized causal pathway follows: HDDS → Iron intake and bioavailability → Ferritin → sTfR → Hemoglobin. This guided model building and ensured that only appropriate confounders were adjusted for.

Demographic, socioeconomic, and clinical data were collected through structured questionnaires and examinations. Trained Anthropometric measurements assessments (height and weight) were obtained using calibrated Seca instruments nearest 0.1 cm by the Seca Hamburg, Germanystadiometer, and the participant stands upright, barefoot, and is positioned according to the Frankfurt plane. Weight is measured to the nearest 0.1 kg (Seca GmBH & Co Kg, Hamburg, Germany) with participants in light clothing and no shoes, following standardized procedures. Each measurement was taken twice and averaged to ensure accuracy. Blood samples were collected for hemoglobin, red blood cell count, mean corpuscular volume, soluble transferrin receptor (sTfR), and ferritin analyses.

### Statistical analysis

Statistical analyses were performed using Stata, version 18. Because women in this study were sampled within family clusters (the mother and her close relatives), we accounted for potential intra-cluster correlation by using cluster-robust standard errors at the household (family) level. This adjustment ensures that the estimated standard errors are not underestimated and that statistical significance is not overstated due to shared environmental and genetic factors within families. All regression models—both linear and logistic—were estimated using the “cluster()” option in Stata, specifying the household identifier as the clustering variable. Multivariable linear regression models were used to examine the relationship between household dietary diversity and key iron-related outcomes. Two separate models were constructed based on the hypothesized causal pathway ( Figure 4). **Model 1** assessed the association between the household dietary diversity score (HDDS) and hemoglobin (Hb), adjusting for sociodemographic and nutritional confounders but *excluding soluble transferrin receptor (sTfR)*, as sTfR is a mediator on the pathway from diet diversity to hemoglobin. This separation was necessary to avoid overadjustment bias, which can attenuate the estimated association between HDDS and Hb. **Model 2** examined the association between HDDS and sTfR as an outcome, to evaluate whether dietary diversity influences tissue-level iron demand. To extend the same modeling framework to a clinically relevant outcome, a third logistic regression model was estimated using anaemia (Hb < 10 g/dL) as the dependent variable. This model replicated the covariate structure of the linear models to ensure comparability, thereby directly linking continuous biomarker measures (sTfR, ferritin) with the binary anaemia outcome. Model fit was assessed using the Akaike Information Criterion (AIC) and Bayesian Information Criterion (BIC), with significance set at p < 0.05. A biplot was used to explore relationships between dietary diversity components and study variables visually. Three regression models were estimated using cluster-robust standard errors at the household level: (1) sTfR ~ HDDS + controls; (2) Ferritin ~ HDDS + controls; and (3) Haemoglobin ~ (sTfR, HDDS) + controls. Ferritin was treated as an outcome rather than a control variable to reflect its biological role in iron metabolism. All models adjusted for age, education, BMI, and socioeconomic status.

## Results

The study included 479 Adivasi women, primarily from the Soliga tribe, with a mean age of 30.91 years (SD = 11.229: 18–65 years). The descriptive statistics are provided in Table 1. Most women were young, with a mean age of 30.9 years (SD = 11.229), with a mean hemoglobin (Hb) level of 11.425 g/dL (SD = 1.709). Red blood cell count (RBC) averaged 4.9 million cells/μL (SD = 0.609), while the mean corpuscular volume (MCV) averaged 70.543 fL (SD = 9.093). Soluble transferrin receptor (sTfR) levels were highly variable, with a mean of 10.432 mg/L (SD = 65.134) and a range of 0.72 to 966 mg/L. Similarly, iron ferritin levels (Ferritin) were also highly variable, with a mean of 56.259 μg/dL (SD = 61.689). HDDS. Other anthropometric and household characteristics included a mean household dietary diversity (HDDS) score of 8.575 (SD = 2.978) among 327 households, a mean weight of 43.778 kg (SD = 9.588), and mean height of 149.492 cm (SD = 15.04). Among the categorical variables, 69.5% of participants reported education levels below pre-university college (PUC), and 73.3% were homemakers. Regarding reproductive history, most women reported one or two pregnancies (53.2% with one pregnancy and 38.8% with two pregnancies). Abortions were reported by 9.78% of participants, while stillbirths were reported by 7.95%. The majority of participants reported one or two live births (43.1% and 39.1%, respectively).

**Table 1.** Characteristics of the Sample Included in the Study.

Variable	N	Mean ± SD or n (%)
Age (Years)	479	30.91 ± 11.2
Hemoglobin (Hb) g/dL	313	11.42 ± 1.7
Red Blood Cells (RBC) 10⁶/µL	313	4.95 ± 0.6
Mean Corpuscular Volume (MCV) fL	311	70.54 ± 9.0
Serum Transferrin Receptor mg/l (sTfR)	218	10.43 ± 65.13
Ferritin ng/ml	218	56.25 ± 61.68
Weight kg	473	43.77 ± 9.58
Height cm	473	149.49 ± 15.04
Household Dietary Diversity (HDDS) score	327	8.57 ± 2.97
Education - No formal education	333	69.5%
Education - Formal education	146	30.5%
Occupation - Others	128	26.7%
Occupation - Housewife	351	73.3%
Gravidity - 1	115	35.2%
Gravidity - 2	127	38.8%
Gravidity - 3	62	19.0%
Gravidity - 4	18	5.5%
Gravidity - 5	4	1.2%
Gravidity - 6	1	0.3%
Parity - 1	133	40.7%
Parity - 2	124	37.9%
Parity - 3	54	16.5%
Parity - 4	11	3.4%
Parity - 5	4	1.2%
Parity - 6	1	0.3%
Abortion - 0	295	90.2%

The aggregation of food groups used to construct the HDDS is presented in Supplementary Table S1.

The mean HDDS score was 8.575 (SD = 2.978), indicating moderate food diversity among the households surveyed. All households (100%) reported consuming cereals, reflecting their role as a dietary staple. The majority also consumed white roots and tubers (88.4%), vitamin A-rich vegetables and tubers (76.1%), and vitamin A-rich fruits (63.9%). Both organ and flesh meats were consumed by 66.1% of households, reflecting occasional inclusion of foods such as chicken, mutton, or beef in their diets. In addition, 74.0% reported egg consumption, and 52.9% consumed fish and other seafood. and legumes, nuts, and seeds were consumed by 72.5%. Milk and milk products were reported by 51.7% of households, reflecting lower levels of dairy consumption compared to other food groups. Oil and fats (70.0%), sweets (70.9%), and spices, condiments, and beverages (70.9%) were also widely consumed, indicating a broad inclusion of diverse food items in the diet. The beverages primarily included tea, coffee, and locally prepared herbal or jaggery-based drinks commonly consumed in the community ( Table 2).

**Table 2.** Composition of the HDDS Score.

Component	Score/ Category	Frequency (Percentage)
HDDS Score	8.575 (2.978)	-
Cereals	Yes	327 (100.0%)
White tubers and roots	No	38 (11.6%)
	Yes	289 (88.4%)
Vegetables ¹	No	78 (23.9%)
	Yes	249 (76.1%)
Fruits ²	No	118 (36.1%)
	Yes	209 (63.9%)
Meat ³	No	111 (33.9%)
	Yes	216 (66.1%)
Eggs	No	85 (26.0%)
	Yes	242 (74.0%)
Fish and other seafood	No	154 (47.1%)
	Yes	173 (52.9%)
Legumes, nuts and seeds	No	90 (27.5%)
	Yes	237 (72.5%)
Milk and milk products	No	158 (48.3%)
	Yes	169 (51.7%)
Oils and fats	No	98 (30.0%)
	Yes	229 (70.0%)
Sweets	No	95 (29.1%)
	Yes	232 (70.9%)
Spices, condiments, and beverages	No	95 (29.1%)
	Yes	232 (70.9%)

¹ The vegetable food group combines vitamin A-rich vegetables and tubers, dark green leafy vegetables, and other vegetables. ² The fruit group is a combination of vitamin A-rich fruits and other fruits. ³ The meat group is a combination of organ meat and flesh meat.

Most participants in both groups were from the Soliga tribe, with no significant differences in tribal distribution ( *p* = 0.35). Marital status, educational status, and BMI were also similar across groups, with no significant differences observed. Additionally, health-related metrics such as Hb, MCV, and ferritin showed no significant differences ( *p* > 0.1). Significant differences were observed across taluks ( *p* < 0.001), with a higher proportion of participants from **Hanur (63.4%)** belonging to the high-diversity group, whereas those from **Chamarajanagar (32.3%)** and **Yelandur (32.3%)** were more commonly in the low-diversity group. Blood relations also differed significantly ( *p* < 0.001), with more participants in the lower diversity group reporting “Another blood relative” (30.8%) or “Second cousin” (36.9%) compared to the “diversity = 0” group. Furthermore, the median sTfR was significantly higher in the group with lower food diversity (5.23) than in the more diverse group (4.49, *p* = 0.041) ( Table 3).

**Table 3.** Characteristics of the population based on low diversity (less than a score of 7) compared to others with a higher diversity score.

Factor	Level	High Diversity N = 262	Low Diversity N = 65	*p*-Value
Subtribe	Betta Kuruba	1 (0.4%)	0 (0.0%)	0.35
	Jenu kuruba	9 (3.4%)	5 (7.7%)	
	Kadu kuruba	4 (1.5%)	2 (3.1%)	
	Soliga	248 (94.7%)	58 (89.2%)	
Marital status	Married	260 (99.3%)	65 (100%)	0.19
	Widowed	2 (0.8%)	0 (0.0%)	
Occupation	Works in a shop but is not earning	262 (100.0%)	65 (100.0%)	
	Self-employed	262 (100.0%)	65 (100.0%)	
	Self-employed agriculture	262 (100.0%)	65 (100.0%)	
Taluk	Chamarajanagar	17 (6.5%)	21 (32.3%)	<0.001
	Gundlupet	38 (14.5%)	15 (23.1%)	
	Hanur	166 (63.4%)	6 (9.2%)	
	Kollegal	19 (7.3%)	2 (3.1%)	
	Yelandur	22 (8.4%)	21 (32.3%)	
Age (years), median (IQR)		24 (22, 28)	24 (23, 29)	0.54
Blood relationship with husband	Another blood relative	19 (7.3%)	20 (30.8%)	<0.001
	First cousin on my father's side	46 (17.6%)	4 (6.2%)	
	First cousin on my mother's side	47 (17.9%)	7 (10.8%)	
	Not a relative	86 (32.8%)	8 (12.3%)	
	Second cousin	62 (23.7%)	24 (36.9%)	
	Uncle	2 (0.8%)	2 (3.1%)	
BMI, median (IQR)		18.8 (17.11, 20.83)	19.3 (17.58, 22.01)	0.29
Education	0	31 (11.8%)	10 (15.4%)	0.44
	1	231 (88.2%)	55 (84.6%)	
Housewife	0	7 (2.7%)	0 (0.0%)	0.18
	1	255 (97.3%)	65 (100.0%)	
Marital status	Married	260 (99.3%)	65 (100%)	0.19
	Widowed	2 (0.8%)	0 (0.0%)	
MCV, median (IQR)		69.6 (64.8, 75.65)	71.25 (67.4, 77)	0.21
Electricity	0	44 (16.8%)	15 (23.1%)	0.24
	1	218 (83.2%)	50 (76.9%)	
Height (cm), median (IQR)		150.45 (147, 154.5)	150.6 (147.7, 153.7)	0.9
Weight (kg), median (IQR)		42.8 (38.4, 48)	44.6 (38.7, 49.8)	0.24
Gravida	1	94 (35.9%)	21 (32.3%)	0.83
	2	99 (37.8%)	28 (43.1%)	
	3	52 (19.8%)	10 (15.4%)	
	4	13 (5.0%)	5 (7.7%)	
	5	3 (1.1%)	1 (1.5%)	
	6	1 (0.4%)	0 (0.0%)	
Parity	1	107 (40.8%)	26 (40.0%)	0.53
	2	97 (37.0%)	27 (41.5%)	
	3	47 (17.9%)	7 (10.8%)	
	4	7 (2.7%)	4 (6.2%)	
	5	3 (1.1%)	1 (1.5%)	
	6	1 (0.4%)	0 (0.0%)	
Hemoglobin (g/dL), median (IQR)		11.5 (10.35, 12.7)	11.35 (10.5, 12.3)	0.33
Ferritin, median (IQR)		34.9 (16.33, 80.6)	28 (15.24, 51.57)	0.16
sTfR, median (IQR)		4.49 (3.36, 7.13)	5.23 (4.16, 8.36)	0.041
MCV, median (IQR)		69.6 (64.8, 75.65)	71.25 (67.4, 77)	0.21

**Note: IQR = Interquartile Range.**

While the multivariable linear regression indicates a statistically significant positive association between ferritin and hemoglobin levels (Coefficient = 0.007, 95% CI [0.003, 0.010], *p* < 0.001), ( Table 4). Ferritin was excluded as a control variable and treated as an outcome to reflect its biological role in the iron metabolism pathway. Accordingly, the analytical framework comprised three related models—sTfR ~ HDDS, ferritin ~ HDDS, and Hb ~ (sTfR, HDDS)—each adjusted for the same covariates (age, education, BMI, and socioeconomic status). Although the ferritin model was not estimated separately in this paper, it was conceptually retained to maintain theoretical coherence across analyses. This refinement enhances interpretability and provides a clearer understanding of how dietary diversity influences iron biomarkers and hemoglobin levels.

**Table 4.** Multivariable Linear Regression of Hemoglobin (Hb) and soluble transferrin receptor (sTfR) as outcomes, with Household Dietary Diversity Score (HDDS) as the main predictor.

Predictor/Variable	Coef.	Std. Err.	t-value	*p*-value	95% Confidence Interval	Sig.
***Hb as Outcome (Model 1)***						
HDDD_Score	0.04	0.10	0.43	0.66	-0.16 to 0.25	
Diversity (1 - Base 0)	0.03	0.38	0.10	0.92	-0.72 to 0.80	
Diversity 2	0.27	0.62	0.43	0.66	-0.95 to 1.49	
Diversity 3	0.30	0.89	0.34	0.73	-1.45 to 2.05	
Ferritin	0.007	0.002	3.86	0.00	0.003 to 0.01	***
Age	-0.01	0.02	-0.38	0.70	-0.067 to 0.04	
Education	0.06	0.04	1.45	0.14	-0.02 to 0.15	
BMI	0.06	0.03	1.98	0.04	0.000 to 0.13	**
Socioeconomic Status	0.84	0.49	1.71	0.08	-0.12 to 1.82	*
Constant	8.95	1.69	5.27	0.00	5.60 to 12.30	***
Predictor/Variable	Coef.	Std. Err.	t-value	*p*-value	95% Confidence Interval	Sig.
***sTfR as Outcome (Model 2)***						
HHFD_Score	-2.97	1.47	-2.02	0.05	-5.88 to -0.00	**
Age	-1.24	1.22	-1.02	0.30	-3.66 to 1.166	
Parity	7.92	5.91	1.34	0.18	-3.73 to 19.58	
Education	-2.54	1.36	-1.87	0.06	-5.23 to -0.14	*
BMI	-4432.47	13553.88	-0.33	0.74	-31150.11 to 22285.16	
Constant	78.77	41.04	1.92	0.05	-2.12 to 159.68	*

Note: *** p < 0.01, ** p < 0.05 (statistically significant), * p < 0.1 (trend only, not statistically significant). The model is adjusted for confounders, including age, education, BMI, socioeconomic status, dietary intake, nutritional markers, etc. To avoid overadjustment bias, soluble transferrin receptor (sTfR) was excluded from the hemoglobin (Hb) model since it lies on the causal pathway between household dietary diversity and hemoglobin (see Figure 4). Separate models were estimated for Hb and sTfR as outcomes.)

Multiple potential confounders are included in the Methods section. However, in the final multivariable regression model ( Table 3), the authors adjusted for a subset of these variables based on the DAG approach, ensuring that only the most relevant confounders were retained to minimize bias while avoiding overadjustment. This selection aligns with the analytical framework and the theoretical considerations guiding the study. The multivariable linear regression analysis shows that HDDS_Score negatively associates with sTfR, the dependent variable ( *β* = −2.975, *p* = 0.045), indicating that higher HDDS_Score values were associated with lower sTfR. Education also exhibited a negative association ( *β* = −2.547). Although Socioeconomic Status showed a weak positive trend (p = 0.08), this did not reach statistical significance (p > 0.05) and is interpreted as a non-significant trend. Other predictors were not statistically significant (p > 0.1) ( Table 4). In the statistical analysis, sTfR was treated as a continuous variable rather than being categorized into levels. Additional bivariate analyses were conducted to identify which food groups within the Household Dietary Diversity Score (HDDS) were associated with soluble transferrin receptor (sTfR) levels. Consumption of **green leafy vegetables**, **meat/organ meat**, and **vitamin C–rich fruits** was linked to significantly lower mean sTfR levels (p < 0.05), indicating improved tissue iron status. Other food groups, including **legumes** and **dairy products**, showed no significant associations. These findings suggest that iron- and vitamin C–rich foods primarily drive the inverse relationship between dietary diversity and sTfR. The logistic regression model assessed the association between sTfR and anaemia as a binary outcome (Hb < 10 g/dL), ( Table 5) with the odds ratio reflecting the increased likelihood of anaemia for each unit increase in sTfR.

**Table 5.** Multivariable Logistic Regression for Anaemia (Hb <10 g/dL) with sTfR as Predictor.

Variable	OR	*p*-value	95% Confidence Interval	Significance
sTfR	1.68	0.000	(1.40, 2.01)	***
Education (Reference: No Education)				
— Primary Education	1.006	0.99	(0.22, 4.53)	
— Secondary Education	1.33	0.73	(0.26, 6.75)	
— Higher Education	0.73	0.70	(0.14, 3.76)	
Ferritin	0.99	0.40	(0.97, 1.00)	
Age	0.96	0.62	(0.80, 1.11)	
BMI	0.93	0.38	(0.79, 1.09)	
Education (Continuous)	1.02	0.84	(0.82, 1.27)	
Constant	0.04	0.23	(0.00, 7.05)	

Significance levels: *** p < 0.01, ** p < 0.05, * p < 0.1.

Although the ferritin level of 56 ng/mL is within the normal range and might suggest adequate iron stores, logistic regression results showed a significant association between sTfR and anaemia (Hb < 10 g/dL)—with an odds ratio of 1.68 (95% CI: 1.404–2.019, *p* < 0.01)—indicating that sTfR is capturing functional iron deficiency or increased erythropoietic activity not reflected by ferritin alone. sTfR levels rise in response to cellular iron demand and tissue iron insufficiency, which can occur even when ferritin is normal, particularly in conditions of chronic disease or inflammation. Therefore, the strong association likely reflects a more sensitive detection of iron-restricted erythropoiesis, supporting the utility of sTfR as a complementary marker in identifying early or functional iron deficiency contributing to anaemia. Other covariates, including age, education, BMI, and ferritin, did not show a statistically significant association with anaemia, as reflected by their non-significant *p*-values ( *p* > 0.05) and odds ratios close to 1.

**Figure 3.**
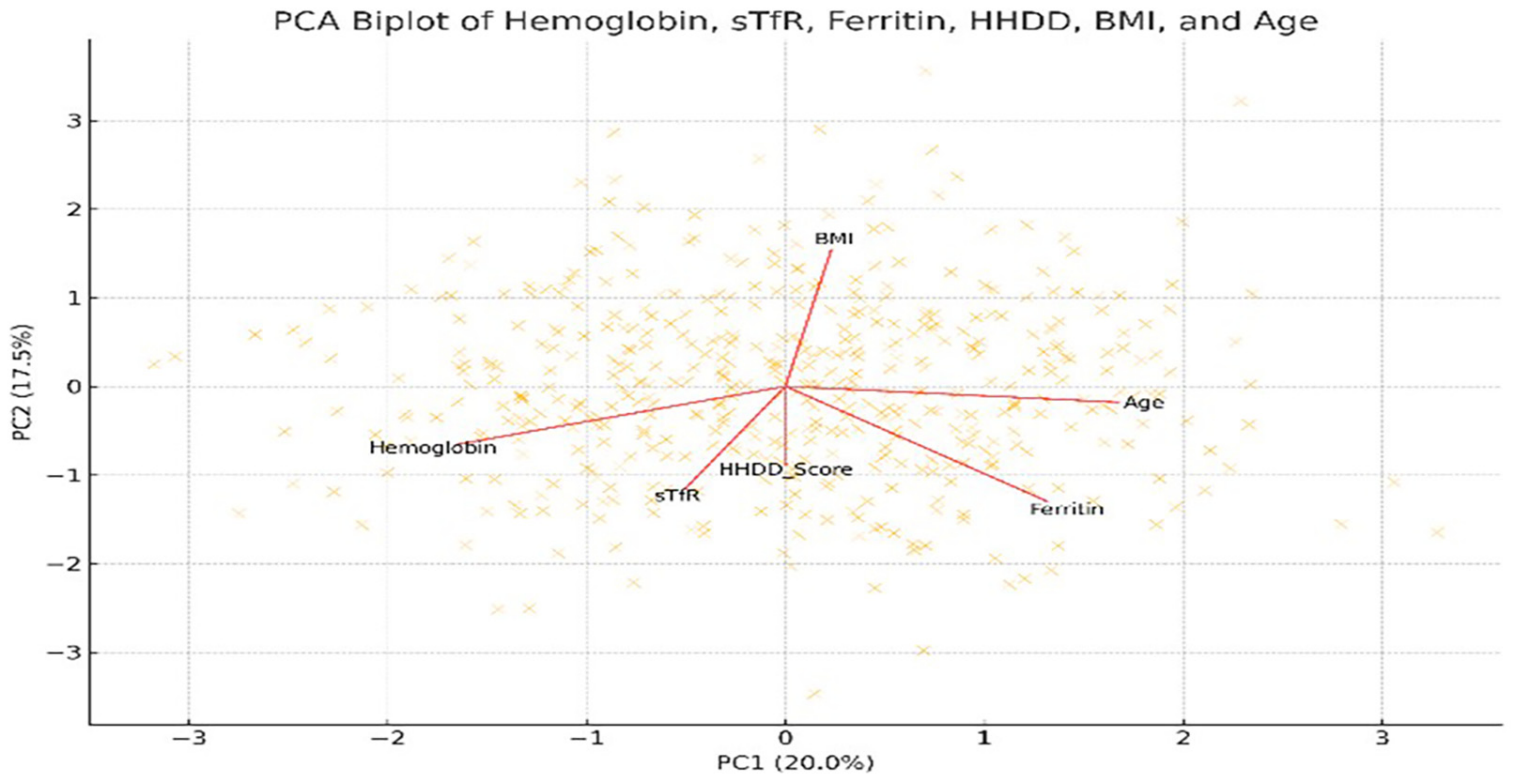
Principal Component Analysis (PCA) biplot of dietary, biochemical, and demographic variables among Adivasi women in the chiguru cohort.

The biplot illustrates ( Figure 3) the relationships among hemoglobin, ferritin, soluble transferrin receptor (sTfR), household dietary diversity score (HDDS), body mass index (BMI), and age. Arrows represent variable loadings on the first two principal components. Opposing directions of HDDS and sTfR reflect their inverse relationship observed in regression analysis. Hemoglobin and ferritin cluster closely, indicating shared patterns in iron status markers.

**Figure 4.**
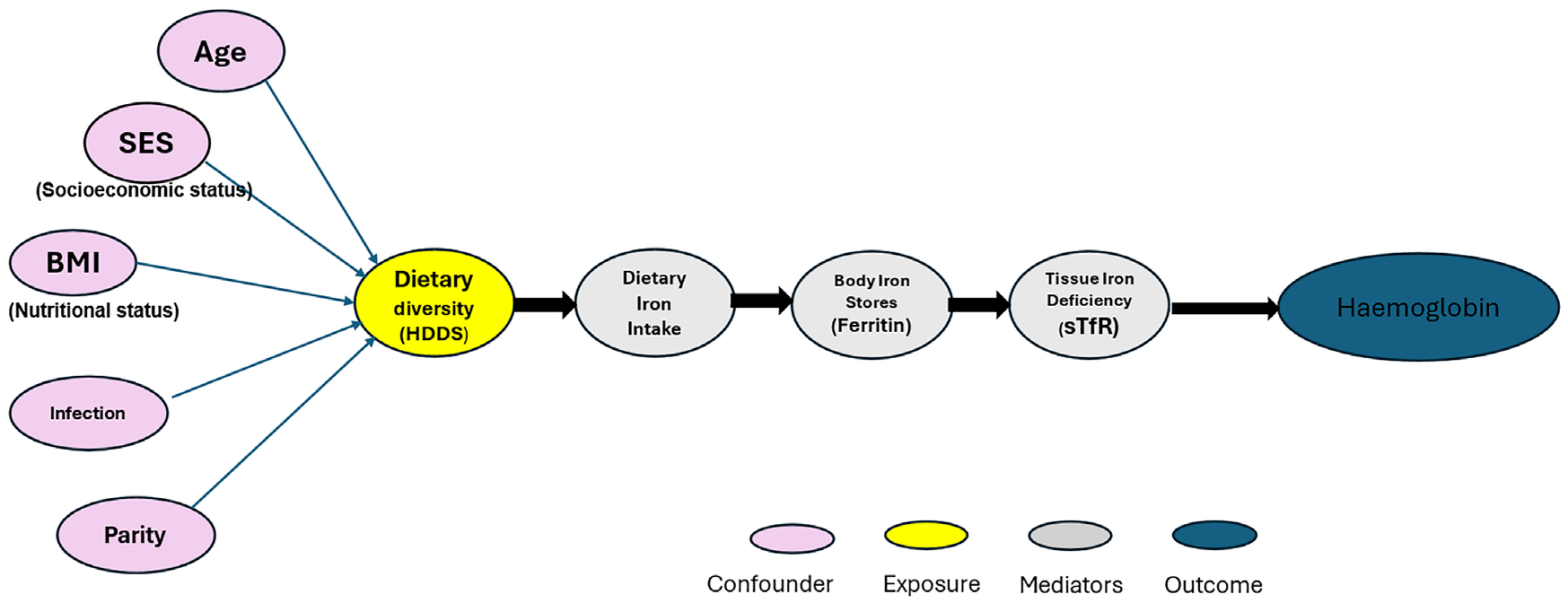
Directed acyclic graph (DAG) illustrating the hypothesized causal pathway linking household dietary diversity (HDDS) to anaemia among Adivasi women in southern Karnataka.

## Discussion

The study reveals a complex relationship between dietary diversity, iron metabolism, and anaemia risk among Adivasi (Indigenous tribal) women. In many remote Indigenous communities, By replicating the modeling framework with anaemia as the outcome, the study strengthens the interpretive link between continuous biomarkers (sTfR, ferritin) and a clinically meaningful endpoint. The consistency of direction and significance across models—particularly the strong association between elevated sTfR and higher odds of anaemia—supports the robustness of our findings. This alignment confirms that the biomarker-based model meaningfully reflects real-world anaemia risk, enhancing its translational relevance for community-level interventions. This study specifically examined non-pregnant women of reproductive age living in Adivasi communities. Understanding anaemia in this population is crucial, as they experience a high burden of nutritional anaemia outside pregnancy. Similar findings have been reported among non-pregnant women in India, linking anaemia to dietary diversity and socioeconomic factors ^3, 4, 20^ Anaemia screening traditionally relied solely on hemoglobin measurements due to limited laboratory facilities and indicators influenced by factors beyond iron status ^27^. It is important to recognize that ferritin, while a widely used indicator of iron stores, is also an acute-phase reactant that can increase in response to inflammation or infection. Because CRP or other inflammatory markers were not measured in this study, the observed association between ferritin and hemoglobin may be influenced by unmeasured inflammation. Including markers such as CRP in future analyses would strengthen the validity of the findings and clarify the ferritin–hemoglobin relationship ^13, 16^. Reliance on this measure alone obscures the underlying aetiology of anaemia. The use of serum ferritin and sTfR in this study improves iron deficiency prediction in vulnerable populations, particularly where infections and malnutrition coexist.

Seasonal variation likely influenced the observed diversity and iron status, as Adivasi diets rely on forest produce such as wild greens, fruits, and tubers, available mainly in certain months. Households surveyed during lean agricultural seasons may therefore have reported lower dietary diversity scores. Although data collection spanned multiple seasons, intra-annual variation was not systematically controlled, warranting longitudinal assessment in future studies. Monotonous staple-based diets high in phytates and low in bioavailable iron elevate anaemia risk.

Among Soliga women, diets are typically centered around staples such as ragi (finger millet) and rice, complemented by small quantities of pulses and seasonal forest produce. Green leafy vegetables, tubers, and wild fruits—particularly amaranthus, drumstick leaves, bamboo shoots, and colocasia—are consumed mainly during the monsoon and post-harvest seasons. Consumption of animal-source foods such as eggs, small fish, and poultry is limited and depends on availability and household income. During lean agricultural periods, reliance on rice and condiments increases, reducing dietary diversity and micronutrient intake. These traditional dietary habits, shaped by forest access, subsistence farming, and cultural norms, influence iron bioavailability and contribute to anaemia risk among Soliga women ^10, 11, 28^.

Although post-partum dietary practices were not directly measured in this study, cultural food restrictions during the post-partum period are commonly reported in Adivasi communities. Such restrictions often involve limiting food quantity or avoiding certain foods, which may temporarily reduce dietary diversity and iron intake. This may partly contribute to the observed dietary patterns and iron status among postnatal women and should be explored in future qualitative and longitudinal studies. Whereas inclusion of animal-source foods and vitamin-C-rich fruits enhances absorption, higher dietary diversity was associated with lower sTfR levels, suggesting improved iron absorption and metabolism.

Whereas inclusion of animal-source foods and vitamin-C-rich fruits enhances absorption. Higher dietary diversity was associated with lower sTfR levels, suggesting improved iron absorption and metabolism. This aligns with known biological mechanisms linking diverse diets—particularly those rich in iron and vitamin C—to reduced tissue-level iron deficiency ^29– 31^. Similar findings across South Africa, Cameroon, and Jharkhand demonstrate that diverse diets correlate with improved iron biomarkers and lower anaemia prevalence ^32, 33^. Together, these findings underscore dietary diversity as a critical determinant of iron metabolism in malnourished settings.

These findings are important for public health, especially for Indigenous communities where anaemia is very common. The average hemoglobin level was 11.42 g/dL, which is below the WHO normal cut-off, showing anaemia of moderate public health concern ^34^. The moderate dietary diversity seen in this group likely limits intake of key nutrients like iron, folate, and vitamin A that are needed to make healthy blood ^35^. Evidence shows that improving dietary diversity can increase hemoglobin levels and reduce anaemia by 20–30% ^36^. Reducing anaemia in these communities needs multiple actions, including improving food diversity through culturally appropriate nutrition programs, strengthening IFA supplementation, and ensuring regular anaemia screening and treatment through the health system ^37^. Involving Indigenous health workers and traditional knowledge can help make these efforts more acceptable and effective ^38^.

Eating many kinds of foods—like grains, pulses, green leafy vegetables, fruits, and animal foods—helps the body get important nutrients such as iron. Vitamin C from fruits and vegetables also helps the body absorb iron better. In Adivasi communities, access to forest foods has reduced, and nutrition problems are common, so iron deficiency is a major concern. Because of this, improving dietary diversity is very important to improve iron levels. , The study found a strong link between high sTfR levels and anaemia. The odds ratio of 1.68 indicates that the likelihood of anaemia increases with higher sTfR levels. This corresponds to a 68% increase in the odds of anaemia compared to the baseline. When expressed as a proportion of the total odds, this equals approximately 62% (calculated as 1.68/(1 + 1.68) × 100). These findings are consistent with earlier studies from Jharkhand’s Indigenous communities, which showed that diets rich in folate were linked to better iron markers such as ferritin, sTfR, and hemoglobin ^1^. Similar evidence comes from studies on anaemia in inflammatory and chronic conditions, where sTfR helps clearly identify iron deficiency anaemia compared to anaemia of chronic disease ^39^. An Indian study also showed that iron supplementation reduced sTfR levels in pregnant women with iron deficiency anaemia, confirming improved iron status ^39^. Other studies, including international research, have shown similar results, demonstrating a strong association between sTfR and iron deficiency status ^28, 40^.

The study findings align with earlier research showing that food security and local agricultural practices strongly influence anaemia prevalence ^41^. Addressing anaemia in Adivasi populations requires targeted, context-specific approaches that go beyond diet alone and include infection control, sanitation improvements, and culturally appropriate supplementation programs ^42^. Strengthening traditional food systems—such as local farming, forest foraging, and Indigenous food preparation practices—can improve access to iron- and vitamin-rich foods while respecting cultural preferences, ecological knowledge, and seasonal availability. Improved dietary diversity, combined with the use of Indigenous foods, has strong potential to reduce iron deficiency and anaemia at the population level. Evidence from homestead food production and nutrition education programs in Bangladesh, Cambodia, Nepal, and the Philippines further supports this approach, demonstrating improvements in dietary diversity and reductions in anaemia prevalence ^43^.

The observed lack of association between dietary diversity and hemoglobin, alongside its significant association with sTfR, is biologically plausible. This distinction is important to note. While haemoglobin defines anaemia clinically, it can be influenced by multiple factors such as infections, inflammation, or haemoglobinopathies. In contrast, sTfR reflects functional iron deficiency and is unaffected by inflammation, thus providing a more specific picture of iron metabolism. Therefore, the observed link between dietary diversity and sTfR indicates improved iron status rather than a direct reduction in anaemia as defined by haemoglobin cut-offs. This lack of association may be due to several interacting factors. Hemoglobin reflects both iron status and non-nutritional influences such as infection, inflammation, and genetic haemoglobinopathies, which are relatively common among Adivasi populations ^19^. In contrast, dietary diversity primarily affects iron absorption and storage over time, processes more sensitively reflected in sTfR than in hemoglobin levels ^15, 27, 30^. Moreover, short-term dietary diversity measures may not capture cumulative dietary intake or seasonal variations in iron consumption ^31, 32^. Together, these factors likely explain why dietary diversity showed a stronger relationship with sTfR but not with hemoglobin. Hemoglobin reflects overall red blood cell concentration, whereas sTfR is a more sensitive marker of iron deficiency at the cellular level, particularly in settings with high infection and inflammation. This underscores the need to integrate newer iron biomarkers, including ferritin, sTfR, and C-reactive protein, into routine community health screenings to distinguish true iron deficiency from inflammation-related anaemia. The cross-sectional design limits causal inference, and recall bias in dietary assessment, along with household-level food data, may not fully capture individual intake. Nevertheless, these findings highlight the value of biomarker-informed surveillance and support future research examining anaemia profiles among men and children to guide more effective and targeted public health interventions.

## Study limitations

While this study provides important insights into anaemia and dietary diversity among Adivasi women, several limitations should be acknowledged. First, the cross-sectional design restricts causal inference between dietary diversity, iron biomarkers, and anaemia; longitudinal follow-up within the cohort will be required to confirm temporal relationships. Second, although dietary data were collected across seasons, intra-seasonal variations and recall bias may have influenced reported dietary diversity ^22, 37^. Third, biochemical markers such as C-reactive protein (CRP) and α-1-acid glycoprotein (AGP) were not measured, limiting the ability to fully adjust ferritin values for inflammation ^13, 30^. Fourth, dietary information was collected at the household rather than the individual level, which may not entirely capture within-household variation in food consumption ^22, 36^. Lastly, the findings are specific to the Soliga and neighboring tribal communities in southern Karnataka and may not be generalizable to all Indigenous populations in India ^11, 41^. These limitations highlight the need for longitudinal and mixed-methods research to better understand causal pathways and contextual factors shaping anaemia in marginalized populations.

## Strengths of the study

To ensure reliable inference, all regression models used cluster-robust standard errors at the family level to account for shared characteristics among related participants. This approach improves the precision and validity of statistical results in clustered data settings.

This study offers several strengths. First, it uniquely combines biochemical iron biomarkers (sTfR and ferritin) with dietary diversity data (HDDS scores), enabling a multidimensional assessment of iron status among Adivasi women—a population underrepresented in the existing literature. Second, the inclusion of sTfR, a markerless effect of inflammation, improves diagnostic specificity for iron deficiency. Third, the study is embedded within an ongoing community cohort Chiguru, ensuring rich contextual data and sustained community engagement. Using culturally appropriate tools and involving Adivasi staff improved the quality of data and made the research more acceptable to the community, following participatory and decolonized research practices.

## Conclusions

This study highlights the critical role of dietary diversity in influencing iron metabolism and the importance of sTfR as a key biomarker for anaemia risk assessment among Adivasi women. While dietary diversity was not directly predictive of hemoglobin levels, its inverse association with sTfR suggests that improving dietary variety can enhance iron status and reduce iron demand at the tissue level. Furthermore, sTfR emerged as a strong predictor of anaemia, reinforcing its potential as a diagnostic tool for targeted interventions in resource-poor settings. Extending the regression model to the binary anaemia outcome further validated the predictive role of sTfR, underscoring its clinical utility alongside dietary diversity indicators.

The findings emphasize the need for culturally relevant strategies to combat anaemia in tribal communities. Promoting the cultivation and consumption of locally available iron-rich foods and encouraging community-led agricultural initiatives can significantly improve dietary diversity. For example, leafy greens such as amaranthus (dantina soppu), drumstick leaves (moringa), colocasia leaves (kesuvina soppu), and wild legumes traditionally gathered or cultivated by Adivasi communities are rich in iron and commonly consumed. Strengthening support for kitchen gardens, seasonal forest-based harvesting, and community seed banks can further empower these populations to preserve their dietary heritage while improving nutritional outcomes. Additionally, strengthening public health interventions by integrating sTfR-based screening and expanding healthcare infrastructure in tribal regions will be essential for early anaemia detection and effective management.

In conclusion, a multidimensional approach that combines nutritional interventions, improved screening protocols, and community engagement is essential for sustainable anaemia reduction. Addressing the underlying barriers will be key to making lasting improvements in nutrition outcomes and overall well-being among Adivasi women. Further research should explore the causes of anaemia and factors driving it across the population level, including men and children, to develop more precise and effective intervention strategies.

Funding: This study was funded by the DBT/Wellcome Trust India Alliance Clinical and Public Health Research Centers grant for the Centre for Training, Research, and Innovation in Tribal Health (CTRITH) [IA/CRC/20/1/600007] awarded to Prashanth NS, Suresh Shapeti, Deepa Bhat, and Upendra Bhojani (support to MK, PS, SS, and TS).

## Institutional Review Board Statement

The Institutional Ethics Committee (IEC) reviewed and approved the study at the Bangalore campus of IIPH-H (Approval number IIPHHB-TRCIEC-216-2021, dated May 6, 2024). Only participants who are willing to participate voluntarily and those who have provided written informed consent are enrolled.

## Informed consent statement

Written informed consent was obtained from all subjects involved in the study. Only women aged 18 years and above were enrolled; no minors participated. For illiterate participants, the consent process included an oral explanation in the local language and witnessed thumbprint consent.

## Abbreviations

The following abbreviations are used in this manuscript:

**Table:** 

HDDS	Household Dietary Diversity
sTfR	Soluble Transferrin Receptor
WHO	World Health Organization
CRP	C-Reactive Protein
AGP	Alpha-1-Acid Glycoprotein
IDA	Iron Deficiency Anaemia
NFHS	National Family Health Survey
FFQ	Food Frequency Questionnaire
FAO	Food and Agriculture Organization
ROC	Receiver Operating Characteristic
AUC	Area Under the Curve
VGKK	Vivekananda Girijana Kalyana Kendra
Hb	Hemoglobin
MCV	Mean Corpuscular Volume
RBC	Red Blood Cell
DAG	Directed Acyclic Graph
BMI	Body Mass Index
IFA	Iron–Folic Acid
ACD	Anaemia of Chronic Disease

## Figures and Tables

**Supplementary Table S1.** 

Question number(s)	Food group	Number of Respondents	Consumed (Yes)	Not Consumed (No)	% Consumed
1	Cereals	272	272	0	100%
2	White tubers and roots	272	237	35	87%
3,4,5	Vegetables ^1^	272	197	74	71.32%
6,7	Fruits ^2^	272	207	65	76.10%
8,9	Meat ^3^	272	222	50	81.61%
10	Eggs	272	192	80	70%
11	Fish and other seafood	272	134	138	50%
12	Legumes, nuts and seeds	272	181	91	66%
13	Milk and milk products	272	123	149	45%
14	Oils and fats	272	176	96	64%
15	Sweets	272	178	94	65%
16	Spices, condiments and beverages	272	177	95	65%

^1^ The vegetable food group is a combination of vitamin A rich vegetables and tubers, dark green leafy vegetables and other vegetables. ^2^ The fruit group is a combination of vitamin A rich fruits and other fruits. ^3^ The meat group is a combination of organ meat and flesh meat.

## Data Availability

The datasets generated and/or analyzed during the current study are not publicly available due to ethical restrictions and protection of participant confidentiality, as required by the Institutional Ethical Review Board of the Indian Institute of Public Health–Bangalore (Approval number IIPHHB-TRCIEC-216-2021, dated 6 May 2024). The IRB specifically mandated that individual-level data containing sensitive health and demographic information from Adivasi women should not be placed in an open repository to prevent risks of misuse or breach of privacy. Data access can be granted upon reasonable request to the corresponding author ( maithili.k@phfi.org) under a data sharing agreement. Access will only be provided to qualified researchers for non-commercial academic purposes, after approval by the Institutional Review Board, and under conditions that ensure data security and participant confidentiality. Extended materials supporting the study (survey tools and summary files) are openly available via Zenodo at: https://doi.org/10.5281/zenodo.17301153 (V.01) Zenodo. Dietary Diversity, Iron Status, and Anaemia Among Adivasi Women: Insights from the chiguru Cohort in Chamarajanagar District, Southern Karnataka. https://doi.org/10.5281/zenodo.17301153 (V1.0) ^44^ This project contains the following underlying data: CTRITH-Cohort-Baseline Questionner-Final_26.04.2022.docx Field_Data_Collection_Checklist (1).pdf Figure 1.jpg Figure 2.jpg Figure 3.jpg PIS-consent from_English.docx PIS-consent_final_kannada.docx Qunatitative-data-sheet.xlsx Data is available under the terms of the Creative Commons Attribution 4.0 International (CC BY 4.0) license. Zenodo repository DOI : https://doi.org/10.5281/zenodo.17301153 (V1.0)
