# Supplementary material for: Dietary Diversity, Iron Status, and Anaemia Among Adivasi Women: Insights from the Chiguru Cohort in Chamarajanagar District, Southern Karnataka
Source: Wellcome Open Res. 2026 Feb 2;10:608. Originally published 2025 Oct 29. [Version 2] doi: 10.12688/wellcomeopenres.24846.2 (PMC13122146; doi:10.12688/wellcomeopenres.24846.2)
Supplement: Peer review history

# Reviewer report: Reviewer response for version 2

**DOI:** 10.21956/wellcomeopenres.28371.r152278

**Published:** 2026-04-27

**Authors:** Anmol Patted

**Affiliations:** 1 Thomas Jefferson University, Philadelphia, Pennsylvania, USA

1. The authors hypothesize that higher HDDS correlates with better iron status in Adivasi women, operationalized as lower sTfR levels and reduced IDA risk. A critical concern arises: sTfR is highly sensitive to the overall rate of erythropoiesis, any condition that increases red blood cell turnover, such as malaria or haemoglobinopathies, can elevate sTfR independently of iron status, rendering it an unreliable standalone marker. This is particularly relevant given that India is a malaria-endemic region and sickle cell trait and beta-thalassemia minor has been reported in the Chamarajnagar district and the Adivasi population in Karnataka. It is unclear whether participants were screened for haemoglobinopathies prior to enrolment.

 The authors are encouraged to explicitly address how conditions elevating erythropoietic demand were accounted for in their analytical framework in the given population. 

 Sickle cell trait and beta-thalassemia minor, both of which produce microcytosis and mild anemia would be misclassified as iron deficiency anaemia on the basis of Hb and sTfR alone. The mean MCV of 70.54 fL is consistent with microcytosis and raises this concern directly. The authors mention hemoglobinopathies briefly in the discussion but do not report MCV-based or RDW-based subgroup analyses that might partially address this.

 2. The study population includes postnatal women and other adult female household members (≥18 years). Postpartum period involves iron recovery dynamics that differ from non-pregnant states, and the paper acknowledges that postnatal women in Adivasi communities frequently observe dietary restrictions that specifically reduce iron-rich food intake (meat, eggs, certain leafy greens) precisely during the window when iron demand is highest. The study must stratify the results by physiological state (postnatal vs. non-pregnant). The authors should also consider testing a formal interaction between postnatal status and HDDS in the sTfR outcome model.

 3. The study states that "Postnatal women were recruited as part of the Chiguru Adivasi family cohort and were followed up at 3 months, 6 months, and 1 year postpartum." Authors need to clarify what data/ samples were collected at each visit, and provide exact n per subgroup and per visit.

 4. The paper enrolls 479 women, yet hemoglobin is available for only 313, and sTfR and ferritin for only 218. How was missing data handled?

 5. It is unclear why authors used Hb < 10 g/dL as the threshold for anemia diagonsis and not the standard WHO guideline. Authors could consider re-running the primary logistic regression in Table 5 using the WHO-recommended threshold of Hb < 12 g/dL for non-pregnant women of reproductive age as the primary outcome, with the current Hb < 10 g/dL analysis retained as a secondary moderate-to-severe anaemia subgroup analysis.

 6. The authors could expand on the study's strength re how community engagement and decolonised research practices were operationalised.

Is the work clearly and accurately presented and does it cite the current literature?

Partly

If applicable, is the statistical analysis and its interpretation appropriate?

I cannot comment. A qualified statistician is required.

Are all the source data underlying the results available to ensure full reproducibility?

Partly

Is the study design appropriate and is the work technically sound?

Partly

Are the conclusions drawn adequately supported by the results?

Partly

Are sufficient details of methods and analysis provided to allow replication by others?

Partly

Reviewer Expertise:

Iron-deficiency/ Iron-deficiency anemia in reproductive age women

I confirm that I have read this submission and believe that I have an appropriate level of expertise to confirm that it is of an acceptable scientific standard, however I have significant reservations, as outlined above.

## References

[1] : Sickle cell allele distribution in southern India: a population-based study in a rural–tribal context. *BMJ Public Health* .2025;3(2) : 10.1136/bmjph-2025-003108 10.1136/bmjph-2025-003108 PMC1250603541069976

[2] : Iron Deficiency in Adults. *JAMA* .2025;333(20) : 10.1001/jama.2025.0452 10.1001/jama.2025.0452 40159291

[3] : Iron indices: What do they really mean?. *Kidney International* .2006;69: 10.1038/sj.ki.5000404 S4-S8 10.1038/sj.ki.5000404 16830699

# Reviewer report: Reviewer response for version 2

**DOI:** 10.21956/wellcomeopenres.28371.r146377

**Published:** 2026-02-12

**Authors:** Suman Chakrabarti

**Affiliations:** 1 International Food Policy Research Institute, Washington, District of Columbia, USA

I am satisfied with the responses.

Is the work clearly and accurately presented and does it cite the current literature?

Yes

If applicable, is the statistical analysis and its interpretation appropriate?

Partly

Are all the source data underlying the results available to ensure full reproducibility?

Partly

Is the study design appropriate and is the work technically sound?

Partly

Are the conclusions drawn adequately supported by the results?

No

Are sufficient details of methods and analysis provided to allow replication by others?

Partly

Reviewer Expertise:

NA

I confirm that I have read this submission and believe that I have an appropriate level of expertise to confirm that it is of an acceptable scientific standard.

# Reviewer report: Reviewer response for version 1

**DOI:** 10.21956/wellcomeopenres.27369.r141104

**Published:** 2026-01-07

**Authors:** Mai-Lei Woo Kinshella

**Affiliations:** 1 Children's & Women's Health Centre of British Columbia, Vancouver, British Columbia, Canada

Thank you for the opportunity to review this paper, which examines iron biomarkers within a population of tribal women in India. Below are some comments for consideration: 

**Major comments:**

*Abstract*

 1. "Geographic variations in dietary diversity among the Soliga community are influenced by forest access, seasonal availability of wild foods, and local agricultural practices" is currently reported in the results but unclear how it was assessed from the methods description. Please elaborate. 

 2. Please rework the conclusions to more reflect the objective of the study, particularly regarding iron status biomarkers which is currently not mentioned in the conclusions. 

*Introduction*

 3. Please define of "tribal communities" as there can be multiple understandings. If this is in reference specifically to India, please explain.

 4.  The "disproportionate impact on tribal communities" is currently not well explained. Which "specific tribal populations" are the authors referring to? Please include the reported rate of anaemia globally, in India and in Indian "tribal communities". 

*Methodology*

 5. "Only women aged 18 years and above" - Please explain if there was a maximum age.

 6. More is needed to explain the HHDD Score methodology. What is the range of scores and how is it derived from the HDDS classifications? How does it extrapolate responses from women to household dietary diversity? How does it relate to the validated Minimum Dietary Diversity for Women measure?

 7. When were blood samples collected? Were blood samples and the dietary diversity questionnaire collected at the same time? 

 8. Any information regarding menstrual cycle during time of blood sample collection? 

 9. Please report using a research checklist such as STROBE. 

*Results*

 10. The full age range needs to be reported. 

 11.  "Oil and fats (70.0%), sweets (70.9%), and spices, condiments, and beverages (70.9%) were also widely consumed, indicating a broad inclusion of diverse food items in the diet" - these are usually unhealthy food groups. What types of beverages? 

 12. Beyond organ meats, other types of meat are currently not reported in text. 

 13. " it is important to note that ferritin is an acute-phase reactant that can be elevated in the presence of inflammation or infection. Given that CRP or other inflammatory markers were not included in the model, the observed association may be confounded by unmeasured inflammatory status. For instance, with a ferritin level of 56 ng/mL, it is unclear whether this reflects adequate iron stores or an inflammatory response. Adjusting for markers such as CRP would strengthen the validity of the findings and help clarify the true nature of the ferritin–haemoglobin relationship" - This is not a study result and is more appropriate for the discussion. 

*Discussion*

 14. "Monotonous staple-based diets, high in phytates and low in bioavailable iron, contribute to high anaemia prevalence" - This study would benefit from more information regarding dietary patterns among Adivasi women, particularly the Soliga tribe,

 15. References in the discussion include studies with school aged children and pregnant women. Please include some references more closely related to your study population - non-pregnant women (of reproductive age? That is currently unclear). 

 16. While the study findings support sTfR as an emerging biomarker of iron status, it does not adequately tease out why dietary diversity was not associated with hemoglobin.

 17. "The authors observed a strong link between elevated sTfR, indicating iron deficiency, and anaemia. The odds of anaemia increased by approximately 68.4% for each unit increase in sTfR, highlighting the critical role of iron deficiency as reflected by high sTfR in the population." Considering that anaemia is defined using hemoglobin cut-offs, dietary diversity is found to be associated with a marker of iron status, but not necessarily anaemia. This needs to be clarified and explained better in the discussion. 

 18. More consideration of study limitations is needed. 

*Overall*

 19. The manuscript would benefit from overall streamlining for conciseness and checking over spelling.

**Minor comments:**

*Abstract*

 1. "the chiguru Adivasi cohort" - should Chiguru be capitalized?

 2. Please provide age range for the 479 women

 3.  HHDD scores...sTfR levels... - please define acronyms on first usage

 4. Please report 95% confidence intervals for the odds ratio.

*Introduction*

 5. *"*Studies have shown that tribal communities often have diets dominated by cereals and starchy staples"  - references needed for these studies

 6. "Protein also plays a role in iron metabolism" Please rework this line for clarity. It currently can be misinterpreted. 

 7.WHO, CRP, AGP...sTfR... - please define acronyms on first usage

*Methodology*

 8. NFHS-5  - please define acronyms on first usage

 9. Please add the HHDD questionnaire as a supplementary file

*Discussion*

*10. "*Indigenous communities" This is the first mention of Indigenous communities. 

Is the work clearly and accurately presented and does it cite the current literature?

Partly

If applicable, is the statistical analysis and its interpretation appropriate?

I cannot comment. A qualified statistician is required.

Are all the source data underlying the results available to ensure full reproducibility?

No

Is the study design appropriate and is the work technically sound?

Partly

Are the conclusions drawn adequately supported by the results?

Partly

Are sufficient details of methods and analysis provided to allow replication by others?

Partly

Reviewer Expertise:

Maternal dietary diversity and pregnancy outcomes

I confirm that I have read this submission and believe that I have an appropriate level of expertise to state that I do not consider it to be of an acceptable scientific standard, for reasons outlined above.

# Response

**Published:** 2026-01-19

**Authors:** maithili Karthik

**Affiliations:** Public health, Public health foundation of India, Bengaluru, Karnataka, India

Major comments: Abstract

**"Geographic variations in dietary diversity among the Soliga community are influenced by forest access, seasonal availability of wild foods, and local agricultural practices" is currently reported in the results but unclear how it was assessed from the methods description. Please elaborate.** Thank you for the comment. We have clarified in the *Abstract* and *Methods* that geographic variations were assessed through stratified analysis of Household Dietary Diversity Scores (HDDS) across taluks and forest proximity.

**Please rework the conclusions to more reflect the objective of the study, particularly regarding iron status biomarkers which is currently not mentioned in the conclusions.** Thank you for the helpful suggestion. We have revised the Abstract conclusion to better align with the study objective by including findings related to iron status biomarkers. The updated text now highlights the inverse association between dietary diversity and sTfR, and the predictive role of sTfR in anaemia.   Introduction

**Please define of "tribal communities" as there can be multiple understandings. If this is in reference specifically to India, please explain.** We thank the reviewer for this valuable suggestion. We have clarified the meaning of “tribal communities” in the introduction to specify that it refers to India’s constitutionally recognized Scheduled Tribes (Adivasi groups), providing cultural and geographical context to the term.

**The "disproportionate impact on tribal communities" is currently not well explained. Which "specific tribal populations" are the authors referring to? Please include the reported rate of anaemia globally, in India and in Indian "tribal communities".** We thank the reviewer for this helpful suggestion. We have now revised the opening paragraph of the Introduction to clarify which tribal populations are referred to (Soliga, Jenu Kuruba, and Kadu Kuruba) and to include anaemia prevalence rates globally, in India, and among Indian tribal communities   Methodology

**"Only women aged 18 years and above" - Please explain if there was a maximum age.** Thank you for this comment. We have clarified in the *Informed Consent* section that there was no maximum age limit; all adult women aged 18 years and above were eligible to participate.

**More is needed to explain the HHDD Score methodology. What is the range of scores and how is it derived from the HDDS classifications? How does it extrapolate responses from women to household dietary diversity? How does it relate to the validated Minimum Dietary Diversity for Women measure?** Thank you for the comment. We have clarified the HHDD methodology in the *Methods – Data Collection* section. The revised text explains that HHDD (range 0–12) was derived from FAO HDDS guidelines, based on the number of food groups consumed in the past 24 hours. Women’s responses were used as reliable household-level proxies, as they manage food preparation. Only the HHDD was used in this study; MDD-W is mentioned for conceptual context

**When were blood samples collected? Were blood samples and the dietary diversity questionnaire collected at the same time?** Thank you for your valuable comment. We have clarified in the *Methodology – Data collection* section that both the dietary diversity questionnaire and blood samples were collected at baseline during the same visit.

**Any information regarding menstrual cycle during time of blood sample collection?** We thank the reviewer for the insightful comment. Information on the menstrual cycle phase during blood collection was not recorded for non-postnatal women.

**Please report using a research checklist such as STROBE.** We thank you for this suggestion regarding the STROBE checklist. Our manuscript has been structured in alignment with the core items of the STROBE guidelines (e.g., clear description of design, setting, participants, variables, and analyses). Therefore, we believe the current version already reflects those principles. However, we will be happy to provide the checklist if the editor deems it necessary.   Results

**The full age range needs to be reported.** Thank you for your comment. We have now added the full age range of study participants in the Results section. For women aged 18 and above, including those aged 18–65 years and other eligible women in the household. The sentence has been revised to report both the mean age and the full range.

 " **Oil and fats (70.0%), sweets (70.9%), and spices, condiments, and beverages (70.9%) were also widely consumed, indicating a broad inclusion of diverse food items in the diet" - these are usually unhealthy food groups. What types of beverages?** Thank you for your insightful comment. We have now specified the types of beverages consumed in the Results section. The revised sentence clarifies that beverages primarily included tea, coffee, and locally prepared herbal or jaggery-based drinks.

**Beyond organ meats, other types of meat are currently not reported in text.** Thank you for this observation. We have now clarified in the Results section that the “meat” group includes both organ and flesh meats (e.g., chicken, mutton, and beef), as indicated in the HDDS food group classification (Table 2).

**" it is important to note that ferritin is an acute-phase reactant that can be elevated in the presence of inflammation or infection. Given that CRP or other inflammatory markers were not included in the model, the observed association may be confounded by unmeasured inflammatory status. For instance, with a ferritin level of 56 ng/mL, it is unclear whether this reflects adequate iron stores or an inflammatory response. Adjusting for markers such as CRP would strengthen the validity of the findings and help clarify the true nature of the ferritin–haemoglobin relationship" - This is not a study result and is more appropriate for the discussion.** Thank you for the helpful suggestion. We agree that the statement about ferritin and inflammation belongs in the Discussion section rather than the Results. We have moved it accordingly and revised the wording for clarity.   Discussion

**"Monotonous staple-based diets, high in phytates and low in bioavailable iron, contribute to high anaemia prevalence" - This study would benefit from more information regarding dietary patterns among Adivasi women, particularly the Soliga tribe,** Thank you for your comment. We have revised the Discussion to include a detailed description of Soliga women’s dietary patterns, supported by relevant literature (references 20, 21, and 41) to provide better cultural and contextual understanding.

**References in the discussion include studies with school aged children and pregnant women. Please include some references more closely related to your study population - non-pregnant women (of reproductive age? That is currently unclear).** We thank you for this valuable suggestion. We have clarified that our study population comprises *non-pregnant women of reproductive age* and added relevant references to similar studies among this group.

**While the study findings support sTfR as an emerging biomarker of iron status, it does not adequately tease out why dietary diversity was not associated with hemoglobin.** Thank you for this constructive feedback. We have now expanded the Discussion to explain the possible reasons for the lack of association between dietary diversity and hemoglobin. We clarify that hemoglobin is influenced by non-nutritional factors such as infection, inflammation, and hemoglobinopathies (15), while sTfR provides a more sensitive reflection of iron absorption and storage (10, 27, 29). We have also noted that short-term dietary diversity measures may not capture seasonal and cumulative dietary patterns (30, 31).

**"The authors observed a strong link between elevated sTfR, indicating iron deficiency, and anaemia. The odds of anaemia increased by approximately 68.4% for each unit increase in sTfR, highlighting the critical role of iron deficiency as reflected by high sTfR in the population." Considering that anaemia is defined using hemoglobin cut-offs, dietary diversity is found to be associated with a marker of iron status, but not necessarily anaemia. This needs to be clarified and explained better in the discussion.** Thank you for this valuable suggestion. We have clarified in the Discussion that dietary diversity was associated with improved iron status (lower sTfR), but not necessarily with anaemia, since haemoglobin levels can be influenced by several non-nutritional factors.

**More consideration of study limitations is needed.** We thank the reviewer for this suggestion. A new subsection titled *“Study Limitations”* has been added at the end of the *Discussion* section, before *Strengths of the Study*, acknowledging the key methodological and contextual limitations of the study.  

**The manuscript would benefit from overall streamlining for conciseness and checking over spelling.** Thank you for this helpful suggestion. We have carefully revised the manuscript throughout to enhance conciseness and clarity. Redundant phrases were removed, and long sentences were shortened, particularly in the Abstract, Introduction (first and fourth paragraphs), and Discussion (paragraphs 2 and 3). We also corrected minor spelling and grammatical errors across the text. These edits have improved the manuscript’s readability without altering the scientific content.  

 Minor comments: Abstract​​​​​​​

**"the chiguru Adivasi cohort" - should Chiguru be capitalized?** Thank you for the observation. The term *chiguru* is intentionally written in lowercase, as it represents the official name of the cohort. We have retained it in lowercase throughout the manuscript for consistency and accuracy.​​​​​​​

**Please provide age range for the 479 women** Thank you for this suggestion. We have now added the age range (18–65 years) in the Abstract to clarify the participants’ demographic coverage.​​​​​​​

**HHDD scores...sTfR levels... - please define acronyms on first usage** Thank you for this suggestion. We have expanded all acronyms (HDDS, Hb, sTfR) upon first mention in the abstract.​​​​​​​

**Please report 95% confidence intervals for the odds ratio.** Thank you for this suggestion. We have now included the 95% confidence interval (1.40–2.01) for the odds ratio as suggested.   Introduction​​​​​​​

**"Studies have shown that tribal communities often have diets dominated by cereals and starchy staples"  - references needed for these studies** Thank you for the suggestion. We have now cited relevant literature (Refs 5, 20, 21) to support the statement on cereal- and starch-based diets among tribal communities.​​​​​​​

**"Protein also plays a role in iron metabolism" Please rework this line for clarity. It currently can be misinterpreted.** Thank you for pointing this out. The line has been revised for clarity to accurately describe the role of protein quality and source in iron absorption.   7.WHO, CRP, AGP...sTfR... - please define acronyms on first usage Thank you for pointing this out. All acronyms (WHO, CRP, AGP, sTfR) have now been expanded at first use in the Introduction for clarity.  ​​​​​​​

**NFHS-5  - please define acronyms on first usage** **Response: **Thank you for the suggestion. The acronym NFHS-5 has now been defined in full as “National Family Health Survey (NFHS-5)” at its first occurrence in the

*Methodology* section. 9 . **Please add the HHDD questionnaire as a supplementary file**

**Response: **Thank you for the observation. The complete Household Dietary Diversity (HHDD) questionnaire has been included as Supplementary File 1 and referenced in the *Data Collection* section.  

**"Indigenous communities" This is the first mention of Indigenous communities.**

**Response: **Thank you for pointing this out. We have clarified the terminology in the Discussion section. The phrase “Indigenous communities” has been revised to “Adivasi (Indigenous tribal) women living in remote communities” to ensure consistency with the rest of the manuscript and accurate representation of the study population.

# Reviewer report: Reviewer response for version 1

**DOI:** 10.21956/wellcomeopenres.27369.r141099

**Published:** 2026-01-05

**Authors:** Suman Chakrabarti

**Affiliations:** 1 International Food Policy Research Institute, Washington, District of Columbia, USA

This is a timely study examining associations between household diet diversity and biomarkers of iron status (Hb and sTfR) in an Indian tribal population. Despite the cross-sectional design, the inclusion of biomarker data is a clear strength. However, several methodological issues limit the credibility of the findings in their current form.

1. **Regression Inference and Standard Errors**
  All regression models should use cluster-robust standard errors at the level of the primary sampling unit, which in this context appears to be the group comprising the mother and her relatives. Failure to account for intra-cluster correlation likely leads to underestimated standard errors and overstated statistical significance.
2. **Use and Presentation of the DAG**
  Although the authors state that a DAG informed their modeling strategy, the DAG is not shown. The DAG should be explicitly presented and should directly justify the choice of covariates included in the regression models. Without this, it is difficult to assess whether the models appropriately address confounding versus overadjustment.
  I think the causal pathway is
  HDDS → Iron intake & bioavailability → Body iron stores (Ferritin) → Tissue iron deficiency (sTfR) → Hemoglobin (Hb)
3. **Model Misspecification and Overadjustment**
  In Table 4, including sTfR and HDDS in the same model predicting hemoglobin is not appropriate. The results suggest that sTfR lies on the causal pathway from diet diversity to hemoglobin. Controlling for a mediator induces overadjustment bias and mechanically attenuates the estimated association between HDDS and Hb toward the null.
4. **Recommended Model Structure**
  A clearer and theoretically consistent approach would be to present three separate models:
  (i) sTfR as a function of HDDS and controls,
  (ii) Hb as a function of sTfR and controls, and
  (iii) Hb as a function of HDDS and controls.
  The same set of theory-informed covariates should be used across all models to maintain interpretability and comparability.
  Drop ferritin from the set of controls and model it as an outcome as well. 
5. **Extension to Anemia Outcomes**
  The same modeling framework should be replicated with anemia as an outcome. This would strengthen interpretation by linking continuous biomarker results to a clinically relevant binary endpoint.
6. **Disaggregation of Diet Scores**
  While diet scores are informative, additional bivariate analyses examining associations between sTfR and individual food groups would help identify which dietary components are driving the observed relationships, improving biological and policy relevance.
  Given the extent of the recommended revisions, a subsequent round of review would be appropriate once these methodological issues are addressed.

Is the work clearly and accurately presented and does it cite the current literature?

Yes

If applicable, is the statistical analysis and its interpretation appropriate?

Partly

Are all the source data underlying the results available to ensure full reproducibility?

Partly

Is the study design appropriate and is the work technically sound?

Partly

Are the conclusions drawn adequately supported by the results?

No

Are sufficient details of methods and analysis provided to allow replication by others?

Partly

Reviewer Expertise:

maternal and child nutrition

I confirm that I have read this submission and believe that I have an appropriate level of expertise to state that I do not consider it to be of an acceptable scientific standard, for reasons outlined above.

# Response

**Published:** 2026-01-19

**Authors:** maithili Karthik

**Affiliations:** Public health, Public health foundation of India, Bengaluru, Karnataka, India

**1. Regression Inference and Standard Errors**

 All regression models should use cluster-robust standard errors at the level of the primary sampling unit, which in this context appears to be the group comprising the mother and her relatives. Failure to account for intra-cluster correlation likely leads to underestimated standard errors and overstated statistical significance.

**Response: **We thank the reviewer for this important observation. We have revised the statistical analysis to include cluster-robust standard errors at the level of the family unit (mother and her relatives) to account for intra-cluster correlation. This revision has been reflected in the “Statistical analysis” and “Strengths of the Study” sections of the manuscript.

**2. Use and Presentation of the DAG**

 Although the authors state that a DAG informed their modeling strategy, the DAG is not shown. The DAG should be explicitly presented and should directly justify the choice of covariates included in the regression models. Without this, it is difficult to assess whether the models appropriately address confounding versus overadjustment.

 I think the causal pathway is

 HDDS → Iron intake & bioavailability → Body iron stores (Ferritin) → Tissue iron deficiency (sTfR) → Hemoglobin (Hb)

**Response: **We thank the reviewer for this helpful comment.. The DAG has now been added as **Figure 4** to the revised manuscript. It depicts the causal pathway (HDDS → Iron intake → Ferritin → sTfR → Haemoglobin) and identifies key confounders (age, BMI, SES, infection, parity). The Methods section has been updated to explain how the DAG guided covariate selection and prevented overadjustment.

**3. Model Misspecification and Overadjustment**

 In Table 4, including sTfR and HDDS in the same model predicting hemoglobin is not appropriate. The results suggest that sTfR lies on the causal pathway from diet diversity to hemoglobin. Controlling for a mediator induces overadjustment bias and mechanically attenuates the estimated association between HDDS and Hb toward the null.

**Response: **We thank the reviewer for this valuable comment. We have addressed this by revising the statistical models to avoid overadjustment. Specifically, **sTfR was excluded from the haemoglobin model**, and **separate models were fitted** for Hb and sTfR as outcomes (changes added in *Statistical Analysis*, and in *Table 4*). The table title and footnote were updated to clearly reflect this change.

**4. Recommended Model Structure**

 A clearer and theoretically consistent approach would be to present three separate models:

 (i) sTfR as a function of HDDS and controls,

 (ii) Hb as a function of sTfR and controls, and

 (iii) Hb as a function of HDDS and controls.

 The same set of theory-informed covariates should be used across all models to maintain interpretability and comparability.

 Drop ferritin from the set of controls and model it as an outcome as well. 

**Response:** We thank the reviewer for this insightful comment. In the revised manuscript, ferritin has been **excluded as a control variable and treated as an outcome**, consistent with its biological role as an intermediate biomarker of iron storage in the causal pathway linking dietary diversity (HDDS) to haemoglobin. Accordingly, three related models were specified:

1. *sTfR ~ HDDS*,
2. *Ferritin ~ HDDS*, and
3. *Hb ~ (sTfR, HDDS)*,
  each adjusted for the same covariates (age, education, BMI, and socioeconomic status).
  Although the ferritin model was not estimated separately in this paper, it was **retained conceptually** to preserve theoretical coherence and improve interpretability. These revisions are reflected in the *Statistical Analysis*, *Results*, and *Discussion* sections. This change aligns the analytical framework with established biological and epidemiological understanding of iron metabolism.

**5. Extension to Anemia Outcomes**

 The same modeling framework should be replicated with anemia as an outcome. This would strengthen interpretation by linking continuous biomarker results to a clinically relevant binary endpoint.

**Response: **We thank the reviewer for this valuable suggestion. In response, We have extended the same analytical framework to include anaemia (Hb < 10 g/dL) as a binary outcome, replicating the covariate structure used in the linear models. This addition enables direct linkage between continuous biomarkers (sTfR, ferritin) and a clinically meaningful endpoint, thereby strengthening the interpretive coherence of our findings.

 The corresponding revisions have been made in the following sections:

- **Statistical Analysis (Methods):** A new paragraph explicitly describes the logistic regression model using anaemia as the dependent variable.
- **Results:** Added clarification that the analytical framework was replicated to test anaemia as a binary outcome (Table 5).
- **Discussion:** Included a paragraph highlighting the robustness and translational relevance of findings across models.
- **Conclusion:** Added a final sentence reinforcing the clinical utility of sTfR when the model is extended to anaemia.

**6.** **Disaggregation of Diet Scores**

 While diet scores are informative, additional bivariate analyses examining associations between sTfR and individual food groups would help identify which dietary components are driving the observed relationships, improving biological and policy relevance.

**Response: **We thank the reviewer for this suggestion. We added **bivariate analyses** examining associations between **sTfR** and individual **food groups** within the HDDS. Consumption of **green leafy vegetables, meat/organ meat, and vitamin C–rich fruits** showed significantly lower sTfR levels (p < 0.05). This addition clarifies which dietary components drive the observed relationship between dietary diversity and sTfR.

# Reviewer report: Reviewer response for version 1

**DOI:** 10.21956/wellcomeopenres.27369.r140169

**Published:** 2025-12-31

**Authors:** Meghan C McCormick

**Affiliations:** 1 University of Miami, Miami, USA

This article is well-written and adds value to the literature in evaluating a largely underserved population. It takes a unique approach in evaluating both biomarkers as well as food diversity as factors related to anemia. There are some areas in which additional information is required to fully understand authors work.

 In general:

 -There were multiple instances throughout the manuscript of acronyms being used without being defined (In abstract-HDDD; In manuscript - sTfR, FAO, FFQ among others). Even with table of acronyms at the end these should be defined clearly the first time that they are used in the text.

 Introduction:

 -In paragraph 2 the same sentence is repeated twice "Low consumption of dairy products, fruits and vegetables...." One sentence should be removed.

 -Additional information regarding what a typical diet consists of would be helpful when stating there is limited dietary diversity.

 Methodology:

 -Please define what is meant by "adivasi women". My understanding is that this is in reference to Indigenous women of India but is not explained in manuscript for readers not familiar with the communities of India. Likewise a brief description of what a "taluk" is would be helpful.

 -After reading it is a bit unclear to me how women were selected for inclusion in this study. A note was made that women were postnatal, were women recruited at postpartum follow-up visits in clinic or hospital setting? What time frame postnatal? When a female was recruited were all female family members over 18 then approached? Some additional detail regarding recruitment is requested.

 -Note made that the "authors now incorporate ferritin for a more appropriate evaluation." Was this lab test incorporated into study later or did all participants have ferritin testing available?

 -More information on DAG requested for those unfamiliar with this approach and the ways in which authors selected factors for inclusion in multivariable analysis

 -Biplot was not mentioned in methods but included in results.

 -Additional information on HHDD score would be appreciated to understand results. For example, what is the range of scores that can be seen and what is considered low, medium or high diversity? Perhaps a supplemental table providing information on how HHDD score is created and interpreted.

 Results:

 -Some of the commentary on interpretation of the data would be better as part of the discussion section. The results should focus on presenting objective data only.

 -The following line "This should be explicitly mentioned in the Statistical analysis section to clarify how sTfR was handled in the model" is likely a comment by the authors which should be removed from the manuscript and addressed in the methods as noted.

 Tables:

 -In table 3 the proportion of individuals residing in each Taluk with high diversity vs low diversity are swapped compared to how the data is presented in the written portion of results. Please make sure data in Table 3 is accurate in each column.

 -I would not consider a p value of >0.05 to be statistically significant and usually would not highlight these results.

 Discussion:

 -The odds ratio of 1.68 cannot be directly converted into percentage odds. To calculate the percentage from the odds ratio, divide the odds ratio by the sum of 1 and the odds ratio, then multiply the result by 100, which works out to ~62%

 -It would be helpful to discuss how seasonal availability in food supply may have affected results. Was data collection spread throughout the calendar year to reflect all seasonal variability in food sources?

 -In some cultures women who are post-partum are restricted in their food intake/variety for a period of time post delivery, is that a potential factor in this population?

Is the work clearly and accurately presented and does it cite the current literature?

Yes

If applicable, is the statistical analysis and its interpretation appropriate?

Partly

Are all the source data underlying the results available to ensure full reproducibility?

Partly

Is the study design appropriate and is the work technically sound?

Yes

Are the conclusions drawn adequately supported by the results?

Yes

Are sufficient details of methods and analysis provided to allow replication by others?

Partly

Reviewer Expertise:

Benign Hematology

I confirm that I have read this submission and believe that I have an appropriate level of expertise to confirm that it is of an acceptable scientific standard, however I have significant reservations, as outlined above.

# Response

**Published:** 2026-01-19

**Authors:** maithili Karthik

**Affiliations:** Public health, Public health foundation of India, Bengaluru, Karnataka, India

In general:

 -There were multiple instances throughout the manuscript of acronyms being used without being defined (In abstract-HDDD; In manuscript - sTfR, FAO, FFQ among others). Even with table of acronyms at the end these should be defined clearly the first time that they are used in the text.

**Response: **Thank you for this important observation. We have now defined all acronyms at their first occurrence in the abstract and main text, including HDDD, sTfR, FAO, and FFQ, while retaining the table of acronyms for reference.

 Introduction:

 -In paragraph 2 the same sentence is repeated twice "Low consumption of dairy products, fruits and vegetables...." One sentence should be removed.

**Response: **Thank you for your observation. We have removed the duplicated sentence in the second paragraph of the Introduction.

 -Additional information regarding what a typical diet consists of would be helpful when stating there is limited dietary diversity.

**Response: **Thank you for your observation. We have added a brief description of the typical diet immediately before discussing low dietary diversity in the Introduction to provide a clearer context.

 Methodology:

 -Please define what is meant by "adivasi women". My understanding is that this is in reference to Indigenous women of India but is not explained in manuscript for readers not familiar with the communities of India. Likewise a brief description of what a "taluk" is would be helpful. **Response: **Thank you for this helpful suggestion. We have clarified the terms *“Adivasi women”* and *“taluk”* at their first mention in the Methods section to ensure clarity for readers who may be unfamiliar with the Indian context.

 -After reading it is a bit unclear to me how women were selected for inclusion in this study. A note was made that women were postnatal, were women recruited at postpartum follow-up visits in clinic or hospital setting? What time frame postnatal? When a female was recruited were all female family members over 18 then approached? Some additional detail regarding recruitment is requested.

**Response: **Thank you for this suggestion **.** We have clarified the recruitment process in the Methods section under Inclusion and Exclusion Criteria, including recruitment of postnatal women, follow-up time points (3 months, 6 months, and 1 year), and inclusion of adult female household members aged 18 years and above.

 -Note made that the "authors now incorporate ferritin for a more appropriate evaluation." Was this lab test incorporated into study later or did all participants have ferritin testing available?

**Response: **Thank you for pointing this out. We have clarified in the Laboratory Assessment section that serum ferritin and sTfR was measured only among postnatal women at baseline during the initial data collection and was not assessed among other adult female household members.

 -More information on DAG requested for those unfamiliar with this approach and the ways in which authors selected factors for inclusion in multivariable analysis Thank you for this. We have added a brief explanation of the DAG approach and how it informed variable selection.

 -Biplot was not mentioned in methods but included in results.

**Response: **Thank you for this. We have now included the biplot description in the methods section.

 -Additional information on HHDD score would be appreciated to understand results. For example, what is the range of scores that can be seen and what is considered low, medium or high diversity? Perhaps a supplemental table providing information on how HHDD score is created and interpreted.

**Response: **Thank you for the suggestion. We have included the food-group aggregation table as a supplementary table and referenced it in the Dietary Assessment section of the Methods (Statistical analysis).

 Results:

 -Some of the commentary on interpretation of the data would be better as part of the discussion section. The results should focus on presenting objective data only.

**Response: **Thank you for this comment. We have revised the Results section to retain only objective descriptive findings and have moved interpretative and explanatory statements to the Discussion section.

 -The following line "This should be explicitly mentioned in the Statistical analysis section to clarify how sTfR was handled in the model" is likely a comment by the authors which should be removed from the manuscript and addressed in the methods as noted.

**Response: **Thank you for pointing this out. We have removed the note from the Results section and clarified in the *Statistical Analysis* subsection that sTfR was treated as a continuous variable in both linear and logistic regression models.

 Tables:

 -In table 3 the proportion of individuals residing in each Taluk with high diversity vs low diversity are swapped compared to how the data is presented in the written portion of results. Please make sure data in Table 3 is accurate in each column.

**Response: **Thank you for this valuable observation. Upon re-examining the dataset, we found that the interpretation of Taluk-level distribution was inadvertently reversed in the text. The table itself was correct, but the description in the Results section was inconsistent with it. We have now corrected the narrative to align with the actual data, which show that a higher proportion of participants from **Hanur (63.4%)** belonged to the **high-diversity** group, whereas those from **Chamarajanagar (32.3%)** and **Yelandur (32.3%)** were more commonly in the **low-diversity** group. This revision ensures consistency between Table 3 and the Results text.

 -I would not consider a p value of >0.05 to be statistically significant and usually would not highlight these results.

**Response: **Thank you for this valuable observation. We agree that results with p > 0.05 should not be interpreted as statistically significant. In response, We have revised the Results section and clarified the interpretation of such findings. Specifically, we now describe these as “non-significant trends” rather than “significant associations.” We have also amended the note under **Table 4** to explicitly state that *p < 0.05* is considered statistically significant, while *p < 0.1* denotes a non-significant trend. Additionally, we added a clarification in the **Discussion** section emphasizing that only associations with *p < 0.05* were treated as significant in this study. Discussion:

 -The odds ratio of 1.68 cannot be directly converted into percentage odds. To calculate the percentage from the odds ratio, divide the odds ratio by the sum of 1 and the odds ratio, then multiply the result by 100, which works out to ~62%

**Response: **We thank you for pointing out this important clarification regarding the conversion of the odds ratio into percentage odds. We have corrected the statement in the Discussion section to accurately reflect the computation. *“The odds of anaemia increased by an odds ratio of 1.68, which corresponds to approximately 62.7% higher odds of anaemia (calculated as 1.68 / [1 + 1.68] × 100 = 62.7%) for each unit increase in sTfR.”*  

 -It would be helpful to discuss how seasonal availability in food supply may have affected results. Was data collection spread throughout the calendar year to reflect all seasonal variability in food sources?

**Response: **Thank you for this valuable observation. We agree that seasonal variation in food availability likely affects dietary diversity and iron status among Adivasi women. In the revised manuscript, I have clarified the temporal coverage of our data collection first para and discussed the potential impact of seasonal food supply on dietary patterns and study findings in the Discussion 3 ^rd^ para. We have also noted this as an important consideration for future longitudinal analyses. -In some cultures women who are post-partum are restricted in their food intake/variety for a period of time post delivery, is that a potential factor in this population?

**Response: **We thank the reviewer for highlighting the role of post-partum dietary practices. We have now added a paragraph in the Discussion section acknowledging that cultural food restrictions during the post-partum period are common in the study population and may contribute to reduced dietary diversity and iron intake. This has been discussed as a potential explanatory factor for anaemia risk.
